# Short-term safety outcomes of mastectomy and immediate implant-based breast reconstruction with and without mesh (iBRA): a multicentre, prospective cohort study

**DOI:** 10.1016/S1470-2045(18)30781-2

**Published:** 2019-02

**Authors:** Shelley Potter, Elizabeth J Conroy, Ramsey I Cutress, Paula R Williamson, Lisa Whisker, Steven Thrush, Joanna Skillman, Nicola L P Barnes, Senthurun Mylvaganam, Elisabeth Teasdale, Abhilash Jain, Matthew D Gardiner, Jane M Blazeby, Chris Holcombe, iBRA Steering Group, Breast Reconstruction Research Collaborative, R Achuthan, I Adwan, S Aggarwal, M Ahmed, M Akelund, D Akolekar, O Al-Jibury, M Amanita, D Appleton, D Archampong, K Asgiersson, R Athwal, A Augusti, S Ayaani, S Bains, B Baker, E Baker, S Baldota, D Banerjee, S Barker, L Barr, P Barry, N Basu, S Bathla, N Bishop, G Boland, O A Branford, R Bright-Thomas, R Brindle, L Brock, V Brown, F Bux, G Byrne, H Cain, L Caldon, M Callaghan, A Carbone, R Carpenter, S Cawthorn, L Chagla, T Challoner, C Chalmers, R Chalmers, S Chambers, M Chana, N Chand, V Chandran, M Chandrashekar, H Charfare, J Chatterjee, S Chatterjee, R Chattopadhyay, A Chaudry, K Chin, K Chong, A Chouhan, C Choy, P Christopoulos, D Clarke, S Clarke, E Clayton, R Clifford, D Cocker, T Collin, N Collis, F Conroy, C Constantinou, A Conway, J Cook, N Coombs, K Cox, A Critchley, M Dakka, M Dani, R Daoud, L Darragh, S Darvesh, I Dash, S Datta, E Davies, S Dawson, E De Sousa, D Debnath, H Deol, H Devalia, R Di Micco, JR Dicks, J Dickson, N Dobner, G Dobson, N Dunne, D Egbeare, D El Sharief, D Elfadl, E Eltigani, D Enver, E Erel, A Evans, G Exarchos, E Fage, H Fatayer, C Fenn, D Ferguson, R Foulkes, J Franks, V Fung, M Galea, T Gandamihardja, A Gandhi, C Garnsey, C Gateley, J Gattuso, S Gawne, N Geerthan, A Ghattura, A Giaramadze, J Gill, AR Godden, S Goh, S Govindarajulu, S Goyal, T Graja, S Granger, M Green, K Grover, G Gui, R Gurung, E Gutteridge, A Hakim, A Halka, W Hamilton-Burke, I Hamo, C Harding-Mackean, A Hargreaves, S Harries, K Harris, P Harris, S Harrison, J Harvey, M Hashem, U Hassan, J Henderson, J Henton, S Hignett, K Hodgkins, K Horgan, S Horn, J Hu, A Hussain, J Iddon, A Iqbal, R Irri, T Irvine, G Irwin, A Iskender, A Ismail, C Ives, K James, R James, N Jiwa, M Jobson, S Joglekar, L Johnson, R Johnson, R Johnson, L Jones, M Ju Hwang, V Kalles, K Kanesalingam, I Karat, M Kaushik, K Kennedy, E Khalifa, H Khan, M Khanbhai, S Khawaja, H Khout, T Kiernan, B Kim, K Kirkpatrick, P Kiruparan, C Kirwan, M Kishore, P Kneeshaw, A Knight, S Kohlhardt, J Krupa, K Krupa, R Kuruvilla, C Laban, LM Lai, I Laidlaw, K Lambert, F Langlands, M Lansdown, N Laurence, S Laws, S Ledwidge, V Lefemine, H Lennon, R Linforth, K Little, A Luangsomboon, J Lund, J Maalo, L MacLennan, RD Macmillan, F MacNeil, TK Mahapatra, E Mallidis, P Mallon, N Manoloudakis, L Maraqa, S Marla, S Masood, J Massey, T Masudi, P Matey, F Mazari, S McCulley, K McEvoy, J Mcintosh, S McIntosh, S McKenzie, P McManus, J McNicholas, I Michalakis, N Mills, G Mitchell, S Monib, M Mullan, C Murphy, G Murphy, J Murphy, B Murthy, S Musa, G Nagra, R Nangalia, S Narayanan, R Nasr, C Navin, R Newton, S Nicholson, N Nuru, R O'Connell, J O'Donoghue, A Ogedegbe, OS Olayinka, S Olsen, G Osborn, C Osborne, H Osman, C Otieno, F Pakzad, A Park, S Parker, P Partlett, A Parvaiz, L Parvanta, G Patel, A Peel, L Peiris, M Pennick, A Peppe, D Perry, S Pilgrim, J Piper, S Poonawalla, E Popa, V Pope, P Pugh, D Rainsbury, K Ramsey, T Rasheed, R Rathinaezhil, T Rattay, D Ravichandran, M Reed, S Refsum, D Remoundos, K Rigby, S Robertson, A Robinson, J Robinson, N Roche, PJ Roy, M Runkel, J Rusby, S Saha, Z Saidan, M Salab, M Saleh, F Salem, A Sami, S Samlalsingh, N Sarfraz, R Shah, S Shaheed, Y Sharaiha, G Shetty, R Shotton, T Sircar, E Skene, S Sloan, B Smith, J Smith, L Soldanova, F Soliman, S Soumian, J Stevens, C Steventon, E Stewart-Parker, T Stringfellow, R Sutaria, R Sutton, H Sweetland, B Swiech, S Tadiparthi, H Tafazal, N Taheri, C Tait, M Tan, S Tang, A Tansley, S Tate, S Tayeh, A Taylor, J Taylor, P Thawdar, C Thomas, S Thomas, S Thomson, A Thorne, R Tillett, Z Tolkien, A Tomlins, A Topps, F Tsang, EJ Turner, P Turton, S Udayasankar, F Ugolini, E Vaughan Williams, R Vidya, B Vijaynagar, R Vinayagam, A Volleamere, V Voynov, S Waheed, T Walker, U Walsh, R Warner, R Waters, A Wilkins, K Williams, G Wilson, M Wiltsher, B Wooler, C Wright, M Wright, L Wyld, M Youssef, C Zabkiewicz, C Zammit, B Zeidan, D Zheng

**Affiliations:** a Bristol Centre for Surgical Research, Population Health Sciences, Bristol Medical School, Bristol, UK; b Bristol Breast Care Centre, North Bristol NHS Trust, Bristol, UK; c Clinical Trials Research Centre, University of Liverpool, Liverpool, UK; d North West Hub for Trials Methodology, University of Liverpool, Liverpool, UK; e Faculty of Medicine, Cancer Sciences Unit, University of Southampton, Somers Cancer Research Building, University Hospital Southampton, Southampton, UK; f Nottingham Breast Institute, Nottingham University Hospitals NHS Trust, Nottingham, UK; g Breast Unit, Worcester Royal Hospital, Worcester, UK; h Department of Plastic Surgery, University Hospitals Coventry and Warwickshire NHS Trust, Coventry, UK; i Nightingale Breast Unit, Manchester University NHS Foundation Trust, Manchester, UK; j New Cross Hospital, Royal Wolverhampton Hospitals NHS Trust, Wolverhampton, UK; k Patient representative, Liverpool, UK; l Department of Plastic Surgery, Imperial College London NHS Trust, London, UK; m Nuffield Department of Orthopaedics, Rheumatology and Musculoskeletal Sciences, University of Oxford, Nuffield Orthopaedic Centre, Oxford, UK; n Linda McCartney Centre, Royal Liverpool and Broadgreen University Hospital, Liverpool, UK

## Abstract

**Background:**

Use of biological or synthetic mesh might improve outcomes of immediate implant-based breast reconstruction—breast reconstruction with implants or expanders at the time of mastectomy—but there is a lack of high-quality evidence to support the safety or effectiveness of the technique. We aimed to establish the short-term safety of immediate implant-based breast reconstruction performed with and without mesh, to inform the feasibility of undertaking a future randomised clinical trial comparing different breast reconstruction techniques.

**Methods:**

In this prospective, multicentre cohort study, we consecutively recruited women aged 16 years or older who had any type of immediate implant-based breast reconstruction for malignancy or risk reduction, with any technique, at 81 participating breast and plastic surgical units in the UK. Data about patient demographics and operative, oncological, and complication details were collected before and after surgery. Outcomes of interest were implant loss (defined as unplanned removal of the expander or implant), infection requiring treatment with antibiotics or surgery, unplanned return to theatre, and unplanned re-admission to hospital for complications of reconstructive surgery, up to 3 months after reconstruction and assessed by clinical review or patient self-report. Follow-up is complete. The study is registered with the ISRCTN Registry, number ISRCTN37664281.

**Findings:**

Between Feb 1, 2014, and June 30, 2016, 2108 patients had 2655 mastectomies with immediate implant-based breast reconstruction at 81 units across the UK. 1650 (78%) patients had planned single-stage reconstructions (including 12 patients who had a different technique per breast). 1376 (65%) patients had reconstruction with biological (1133 [54%]) or synthetic (243 [12%]) mesh, 181 (9%) had non-mesh submuscular or subfascial implants, 440 (21%) had dermal sling implants, 42 (2%) had pre-pectoral implants, and 79 (4%) had other or a combination of implants. 3-month outcome data were available for 2081 (99%) patients. Of these patients, 182 (9%, 95% CI 8–10) experienced implant loss, 372 (18%, 16–20) required re-admission to hospital, and 370 (18%, 16–20) required return to theatre for complications within 3 months of their initial surgery. 522 (25%, 95% CI 23–27) patients required treatment for an infection. The rates of all of these complications are higher than those in the National Quality Standards (<5% for re-operation, re-admission, and implant loss, and <10% for infection).

**Interpretation:**

Complications after immediate implant-based breast reconstruction are higher than recommended by national standards. A randomised clinical trial is needed to establish the optimal approach to immediate implant-based breast reconstruction.

**Funding:**

National Institute for Health Research, Association of Breast Surgery, and British Association of Plastic, Reconstructive and Aesthetic Surgeons.

## Introduction

Up to 40% of the 1·7 million women diagnosed with breast cancer each year will require a mastectomy as the surgical treatment for their disease.^1^, ^2^, ^3^ Mastectomy can profoundly affect body image and self-esteem, and immediate breast reconstruction, performed at the time of mastectomy, is offered to improve quality of life.^4^

Implant-based breast reconstruction is the most commonly performed reconstructive procedure worldwide.^5^, ^6^ Traditionally, a two-stage procedure is done, involving the initial placement of a tissue expander in the subpectoral pocket, sequential expansions with saline until the desired volume is achieved, and replacement of the expander with a fixed-volume implant. The introduction of biological and synthetic meshes have revolutionised this technique. The mesh is sutured between the lower edge of the pectoralis muscle and the chest wall to create a larger subpectoral pocket that can accommodate a fixed-volume implant at the time of the initial surgery. This approach facilitates single-stage direct-to-implant reconstruction without the need for a second procedure, with substantial associated benefits for patients and health-care providers. The mesh can improve cosmetic outcomes by allowing improved lower-pole projection and creating a more natural-looking ptotic result. A range of biological (eg, acellular dermal matrix [ADM]) and synthetic (eg, titanium-coated polypropylene) meshes are available. These meshes differ notably in price and, in the absence of comparative evidence, product selection is largely dependent on surgeon preference.

**Research in context**

**Evidence before this study**

At the start of this study in August, 2013, we searched MEDLINE, Embase, and the Cochrane database using the search terms for implant reconstruction—“[implant$; expander$; prosthe$]” AND “mesh [including ADM; acellular derma$; AlloDerm, SurgiMend, Strattice]”, for studies published between Jan 1, 2000, and Aug 1, 2013—to identify original papers and systematic reviews investigating the outcomes of mesh-assisted implant reconstruction. We identified eight systematic reviews and 61 primary studies, including one randomised controlled trial. All the systematic reviews were of low quality and at high risk of bias. The randomised clinical trial compared two-stage expander-implant reconstruction with and without mesh. It was well designed and at low risk of bias, but was stopped prematurely because of problems with recruitment. The primary outcome was postoperative pain and pain during the expansion period, but not the safety of using mesh. An interim analysis suggested that there was unlikely to be a difference in pain scores between the treatment groups in the randomised controlled trial. The remaining studies comprised 40 comparative studies and 20 case series, all of which were at high risk of bias.

We did another PubMed search in April, 2018, and identified one small randomised clinical trial that compared standard two-stage expander-implant reconstruction with mesh-assisted, single-stage, direct-to-implant breast reconstruction. The frequency of complications in the single-stage, mesh-assisted, direct-to-implant group was significantly higher than that in patients receiving two-stage expander implant reconstruction, and the study was stopped prematurely. A further small randomised clinical trial compared biological and synthetic mesh in single-stage, direct-to-implant breast reconstruction. Although this study provided evidence that a trial might be possible, it was underpowered and insufficiently well designed to produce meaningful results. Several small exploratory trials have compared different types of biological mesh. These studies did not show any differences between products, but were small and underpowered.

**Added value of this study**

This prospective cohort study of more than 2000 patients having immediate implant-based breast reconstruction in the UK provides high-quality, real-world data regarding the short-term safety outcomes of different implant-based techniques with and without mesh. The frequency of key complications, including implant loss, infection, re-admission, and re-operation for complications of reconstructive surgery, was much higher than anticipated; although adverse outcomes were associated with smoking and increased body-mass index, there was no association with the use or type of mesh in this non-randomised study. These findings support the need for a future pragmatic randomised clinical trial to determine the most clinical and cost-effective technique of implant-based breast reconstruction.

**Implications of all the available evidence**

Complications after immediate implant-based reconstruction with and without mesh are high, and patients should be carefully counselled regarding their surgical options. Surgeons should commit to robustly assessing mesh-assisted, implant-based breast reconstruction in the context of a well designed randomised clinical trial. Further work is needed to explore the most acceptable study design and test the feasibility of randomisation in a pilot randomised clinical trial. Urgent work will also be necessary to determine how the high incidences of complications shown in this study could be reduced.

The practice of immediate implant-based breast reconstruction has evolved further with the introduction of so-called muscle-sparing techniques.^7^ These techniques involve wrapping the implant in mesh and placing it on top of, rather than underneath, the pectoralis muscle. This pre-pectoral technique might further improve outcomes for patients by reducing postoperative pain and preventing implant animation, the upward movement of the implant seen when the pectoralis muscle contracts.^7^

Despite the widespread adoption of mesh-assisted techniques into practice, evidence to support the proposed benefits of mesh is lacking.^7^, ^8^, ^9^, ^10^ A multicentre Dutch randomised controlled trial^11^ showed significantly increased numbers of complications in single-stage, direct-to-implant reconstruction with mesh compared with standard two-stage expander-implant techniques. The study was criticised because the participating surgeons had limited experience with the technique,^12^ but further analysis did not identify a learning curve effect.^13^ However, other large multicentre prospective studies have not shown a significant difference in incidence of complications or patient-reported outcomes between single-stage direct-to-implant and two-stage techniques,^14^ or between two-stage expander-implant reconstruction done with and without acellular dermal matrix.^15^ Although these findings are supportive of the technique, there remains the need for high-quality evidence from a randomised study to support practice. A small randomised clinical trial^16^ that compared biological and synthetic mesh in single-stage direct-to-implant reconstruction was underpowered and insufficiently well designed to generate meaningful results.

There is therefore a need for high-quality research to establish the safety and effectiveness of mesh in immediate implant-based breast reconstruction, to determine what mesh should be recommended, and, as practice evolves, to determine if the implant should be placed on top of or underneath the pectoralis muscle.^7^

Randomised clinical trials are ideally needed, but randomised clinical trials in breast reconstruction are challenging because of patient and surgeon preference^17^ and previous trials have closed prematurely because not enough patients were able to be recruited.^18^, ^19^ Careful pre-trial work is therefore needed to ensure that a future randomised clinical trial is well designed and addresses questions that are important to patients and the reconstructive community.

iBRA (implant Breast Reconstruction evAluation)^20^ is a four-phase study that aims to inform the feasibility, design, and conduct of a future trial in immediate implant-based breast reconstruction. Results from phase 1 (a national practice questionnaire to understand current practice) were previously reported.^21^, ^22^ Here we report the primary endpoint for phase 2, a prospective multicentre national study to determine the short-term clinical outcomes of different approaches to immediate implant-based breast reconstruction in the UK and inform the selection of comparators and sample size for a future randomised clinical trial.

## Methods

### Study design and participants

For this prospective, multicentre iBRA study, we invited all UK breast or plastic surgical units performing immediate implant-based breast reconstruction to participate in the study, through the UK Trainee Collaborative Research Network and two professional associations, the Association of Breast Surgery and the British Association of Plastic Reconstructive and Aesthetic Surgeons. 81 centres participated in the study.

Women aged 16 years or older, who had a mastectomy and immediate implant-based breast reconstruction using any technique for malignancy or risk reduction at participating centres, were consecutively recruited to the study. Patients were excluded if they had reconstruction with an implant in combination with a tissue flap (eg, latissimus dorsi flap and implant), had delayed reconstruction, or had revisional surgery.

Ethics approval was not required, as defined by the HRA decision tool. The study involved the collection of clinical and patient-reported outcome data as routinely recommended by guidelines for good practice^23^ and outcomes assessed against these quality standards. Each participating centre was required to obtain local audit approvals and register the study before commencing study recruitment, consistent with the methods of previously reported multicentre prospective trainee collaborative studies. Patient consent was not required for routine clinical data collection, but patients provided written informed consent to receive patient-reported outcome questionnaires, in keeping with the methods employed in the UK National Mastectomy and Breast Reconstruction Audit.^24^ All data were recorded in an anonymised format on a secure web-based database, REDCap.^25^

### Procedures

We prospectively identified eligible patients from clinics, multidisciplinary team meetings, and theatre lists. Simple demographic, comorbidity, and operative data were collected for each participant.

All patients had skin or nipple-sparing mastectomy followed by immediate implant-based breast reconstruction. Implants or tissue expanders could be placed under the pectoralis muscle (subpectoral) with or without biological or synthetic mesh, or on top of the muscle (pre-pectoral) supported by mesh. Since one aim of the study was to explore current practice to inform a future trial, no restrictions were placed on the technique used, but details of the procedures done were recorded.

Participating surgeons did the procedures according to their routine practice. Mesh choice (biological or synthetic and the specific product used), implant selection (definitive fixed-volume implant, adjustable implant, or temporary tissue expander), and implant positioning (pre-pectoral or sub-pectoral) were as per surgeon preference. Strategies to reduce infection (eg, use of laminar flow, glove change before implant insertion, and wound lavage) were as per local policy. Reconstructions were considered to be two-stage if a temporary expander was placed at the time of the initial mastectomy and a second procedure was planned to insert a definitive implant at a later date.

Precise details of the techniques used varied by surgeon, but broadly, for submuscular reconstructions, a tissue expander was inserted in a pocket created under the pectoralis muscle. Serratus fascia could be raised to provide complete expander coverage, or the lateral aspect of the expander could be left subcutaneous as per surgeon preference.

Sub-pectoral reconstruction with mesh involved releasing the lower boarder of the pectoralis muscle from the chest wall and suturing the mesh to the free edge of the muscle. A definitive fixed-volume implant, adjustable implant, or tissue expander was then inserted according to surgeon preference and the mesh either sutured at the level of the inframammary fold or tucked under the implant, depending on the product used. For dermal sling reconstruction, the pectoralis muscle was detached in a similar way and the lower mastectomy flap de-epithelialised and sutured to the free muscle edge to provide coverage of the lower pole of the implant. It is not standard practice to use mesh in this procedure.

Finally, for pre-pectoral reconstruction, the pectoralis muscle was not disturbed, but a fixed-volume implant, adjustable implant, or temporary tissue expander completely or partially wrapped in mesh (depending on surgeon preference and product selection) was placed in the mastectomy cavity and sutured into place.

In all cases, perioperative and postoperative antibiotics and drains were used according to local policy or surgeon preference.

Complication and oncological data were collected by the clinical team at 30 days and 3 months after reconstruction by clinical or case-note review, or both, depending on when the patient returned for follow-up.

Participants were approached in the clinic or during their hospital stay to obtain consent for patient-reported outcome assessment at 3 months after surgery. The patient-reported outcome was a modified version of the 3-month questionnaire used in the UK National Mastectomy and Breast Reconstruction Audit (NMBRA) and included patient self-report of complications occurring in the 3 months after surgery.^24^ Questionnaires were sent centrally by post or e-mail, depending on patient preference, with a reminder sent 1 month after the initial questionnaire if no response was received. Patient satisfaction was also assessed at 18 months after surgery.^20^ Analyses are ongoing and these results will be reported elsewhere.

### Outcomes

On the basis of published national quality standards for breast reconstruction derived from the NMBRA,^23^ we prespecified four key outcomes to assess the short-term safety of different approaches to immediate implant-based breast reconstruction.^20^ We chose these outcomes because it was anticipated that a safety outcome might be the primary endpoint of a future trial and equivalence in a non-randomised study was important in informing the selection of potential comparators for this study. The outcomes were defined implant loss (the unplanned removal or loss of the implant as a result of infection or other complication), infection (the presence of a hot, red breast requiring treatment with antibiotics, surgery, or both), re-admission (any unplanned re-admission to hospital after discharge for any complication of surgery), and reoperation (any return to the operating theatre for a complication within 3 months of the reconstruction procedure). Any implant loss, infection, re-admission, or re-operation occurring at any timepoint within the first 3 months of the initial reconstruction that was assessed by clinical review or patient self-report was considered an event and included in the analysis.

We identified potential risk factors for each of these key outcomes using a prespecified exploratory risk-factor analysis. We identified specific variables of interest a priori from the published literature and expert opinion, and included the following patient-related and procedure-related factors: age, body-mass index (BMI), smoking (current smokers *vs* others), previous radiotherapy to the ipsilateral breast (yes *vs* no), receipt of neoadjuvant chemotherapy (yes *vs* no), bilateral surgery (yes *vs* no), mastectomy type (nipple-sparing *vs* other mastectomy types), type of implant or expander used (fixed-volume *vs* adjustable implants or expanders), and type of immediate implant-based breast reconstruction performed. We classified reconstruction procedures according to the mode of lower pole coverage as being: submuscular or subfascial without mesh; dermal-sling procedures using the patient's own tissue; biological mesh-assisted (including acellular dermal matrix and non-dermal biological products); synthetic mesh-assisted; pre-pectoral if the implant was placed on top of the pectoralis muscle with or without mesh; and other if a combination of techniques was used (eg, dermal sling and mesh).

For quality assurance purposes, the principal investigator at each participating site was asked to independently validate the primary outcomes for all study participants at 3 months and to check complete case ascertainment.

### Statistical analysis

Because we aimed to inform the design of a future randomised clinical trial, the study was powered to establish parameters required for a sample size calculation and to inform further aspects of trial design, such as entry criteria for the future trial.

At the time of study design, four clinical outcomes (implant loss, re-admission, re-operation, and infection) were considered to be potential primary outcomes in a randomised controlled trial, and a wide range of treatment approaches for immediate implant-based breast reconstruction were routinely offered.^22^ A large sample was therefore required to estimate, with reasonable precision, the incidence of the four clinical outcomes of interest (implant loss, re-admission, reoperation, and infection) within treatment approaches, and to determine how implant procedures are performed and any variation in patient selection for each of these approaches. We therefore planned to recruit as many patients as possible and follow all patients to 3 months. The NMBRA^24^ observed that 9% of patients who had immediate implant-based breast reconstruction reported implant loss at 3 months. When designing a full trial, establishing this proportion with reasonable precision would be required. A sample size of 197 participants would allow a two-sided 95% CI for a single proportion, assumed to be 0·09, extending from 0·05 to 0·13, using the large sample normal approximation. Allowing for the 15% loss to follow-up at 3 months reported in the NMBRA, an analysis of implant loss at 3 months required at least 235 patients to be recruited to inform a future trial with implant loss as a primary outcome. To determine how implant procedures are performed, centres participating in a national practice questionnaire (n=81)^21^, ^22^ were eligible to participate. All centres were eligible because each unit was expected to vary in caseload and perform a relatively small number of procedures (4–40 per year). All analyses are based on the number of patients, not the number of implants.

We did the analysis according to a prespecified statistical analysis plan approved by the trial steering group. We used simple summary statistics to describe demographic, procedure, process, and outcome data, overall and by procedure type. We summarised categorical data by counts and percentages, and continuous data by median, upper and lower quartiles, and range. We did no formal statistical testing.

We established the proportion and 95% CI of patients for each of the four key clinical outcomes to compare our findings against those reported in the NMBRA^24^ and published national quality standards.^23^

We did a prespecified risk-factor analysis using multivariable logistic regression. Variables of interest included patient age, BMI, smoking status, previous radiotherapy to the ipsilateral breast, receipt of neoadjuvant chemotherapy, bilateral surgery, nipple-sparing versus other mastectomy types, use of fixed-volume versus adjustable implants or expanders, and type of immediate implant-based breast reconstruction performed.

Data were considered missing at random (appendix p 2) and therefore no missing data items were imputed. We did a complete case analysis. We considered this approach as unlikely to lead to bias because all included risk factors were measured once per patient.^26^ We checked linearity for continuous variables for all four logistic models using locally estimated scatterplot smoothing (LOESS) smoothed-line plots. These checks showed no obvious evidence of non-linearity for the effects of the three continuous variables. We considered a p value of less than 0·05 to be statistically significant, and made no adjustments for multiplicity. Instead, we took into consideration relevant results from other studies in the interpretation of results, and emphasise the exploratory nature of this study. We used SAS (version 9.3) for all analyses.

This study was registered as an International Standard Randomised Controlled Trial, number ISRCTN37664281, and the protocol was published in 2016.^20^

### Role of the funding source

The funder of the study had no role in study design, data collection, data analysis, data interpretation, or writing of the report. The corresponding author had full access to the data in the study and had final responsibility for the decision to submit for publication.

## Results

Between Feb 1, 2014, and June 30, 2016, 2217 records were entered onto the REDCap database. Of these, 109 (5%) were excluded: 34 patients had surgery outside the study period, 22 records did not include an operation date, 50 records provided no information regarding the type of surgery performed, and three patients did not have an immediate implant-based breast reconstruction. 2108 patients were therefore included in the analysis (figure). The median recruitment per unit was 14 cases (IQR 6–39). Further details about unit recruitment are in the appendix (pp 3–7).

**Figure:**
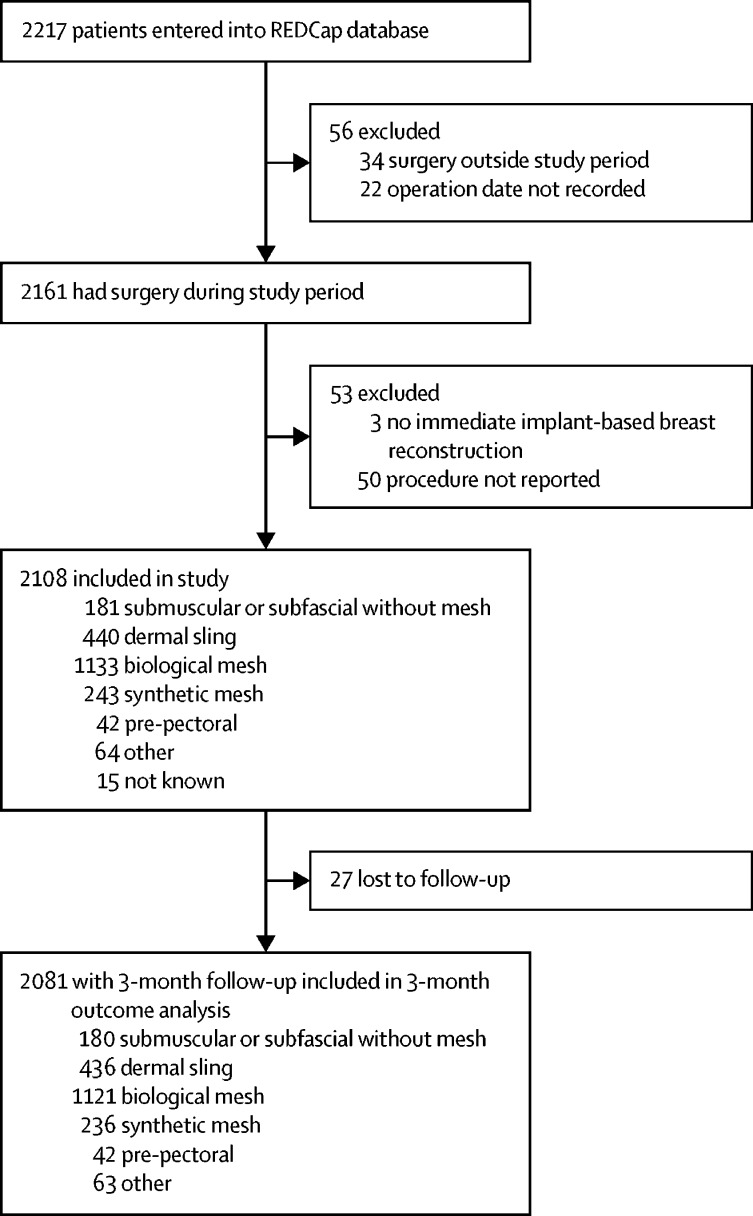
Trial profile For patients with two implant-based reconstructions in which technique differed by breast (n=10), these patients are summarised in both groups, depending on the approach used, and once in the total. Variations of approaches per breast within patient were: biological mesh and dermal sling (n=3), biological mesh and synthetic mesh (n=1), dermal sling and submuscular or subfascial (n=4), other and submuscular or subfascial (n=1), and other and synthetic mesh (n=1).

Biological mesh-assisted reconstruction was the most commonly performed procedure, with 1133 (54%) of 2108 patients undergoing this technique. Fewer patients had synthetic mesh-assisted immediate implant-based breast reconstruction (243 [12%]), and 181 (9%) received traditional subpectoral reconstruction without mesh. Subpectoral reconstruction with a dermal sling was done in approximately a fifth of patients (440 [21%]) and pre-pectoral reconstruction with mesh (42 [2%]) was done in the latter stages of the study. 64 (3%) patients received a combination of techniques (eg, subpectoral reconstruction with dermal sling and mesh) and for 15 (7%) patients, details of the technique were not reported. Strattice (LifeCell, Brachburg, NJ, USA) or SurgiMend (Integra LifeSciences) were used in 1215 (66%) of the 1831 mesh-assisted procedures, but in total, 14 different products were used during the course of the study (Artea, BioDesign, Braxon, Cellis, Meso Biomatrix, Native, Protexa, Silk, Strattice, SurgiMend, TIGF, TiLOOP, Veritas, and XCM), with an increasing variety of products used as the study progressed (data not shown).

The median age of study participants was 49 years (43–57; table 1). Median BMI was at the upper end of the normal range (24·8 kg/m^2^; IQR 22·3–28·2), and was highest overall in patients who had dermal sling reconstruction (table 1). 206 (10%) of 2108 patients were current smokers and 139 (7%) had received previous radiotherapy to the ipsilateral breast (table 1). The 2108 study participants had 2655 implant-based reconstructive procedures. 411 (19%) patients had risk-reducing mastectomies and 1505 (72%) had malignancy only. Of the 547 (26%) patients having bilateral surgery, 188 (34%) had a contralateral risk-reducing mastectomy at the time of their index cancer operation (table 2).

**Table 1:** Patient demographics, by type of implant-based reconstruction

		All patients*(N=2108)	**Submuscular or subfascial**†**(n=181)**	**Dermal sling (n=440)**	**Biological mesh (n=1133)**	**Synthetic mesh (n=243)**	**Pre-pectoral (n=42)**	**Other (n=64)**‡	**Not known (n=15)**
**Age, years**									
Median (IQR; range)		49 (43–57; 16–83);	49 (43–56; 16–80)	51 (45–59; 24–81)	49 (42–56;20–80)	50 (43–58; 20–83)	48 (38–52; 19–73)	49 (43–58; 23–74)	46 (46–52; 39–63)
Not known		13 (<1%)	0	2 (<1%)	4 (<1%)	1 (<1%)	0	0	6 (40%)
**Body-mass index, kg/m^2^**									
Median (IQR; range)		24·8 (22·3–28·2; 14·4–54·0)	24·0 (21·8–27·7; 17–51·6)	28·0 (24·8–32·2; 18·3–54·0)	24·0 (21·9–26·9; 14·4–44·5)	24·8 (22·1–28·0; 17·3–42·6)	23·8 (22·2–27·1; 18·7–36·0)	27·6 (23·8–31·1; 18·0–40·0)	23·8 (23·4–26·5; 18·8–32·2)
Not obese (BMI <30 kg/m^2^)		1613 (77%)	133 (73%)	247 (56%)	976 (86%)	184 (76%)	33 (79%)	44 (69%)	4 (27%)
Obese (BMI ≥30 kg/m^2^)		367 (17%)	34 (19%)	163 (37%)	107 (9%)	36 (15%)	9 (21%)	19 (30%)	1 (7%)
Not known		128 (6%)	14 (8%)	30 (7%)	50 (4%)	23 (9%)	0	1 (1%)	10 (67%)
**Smoking status**									
Non-smoker		1626 (77%)	137 (76%)	335 (76%)	881 (78%)	193 (79%)	31 (74%)	52 (81%)	6 (40%)
Ex-smoker (stopped more than 6 weeks previously)		231 (11%)	19 (10%)	54 (12%)	123 (11%)	22 (9%)	8 (19%)	5 (8%)	0
Current smoker		206 (10%)	22 (12%)	42 (10%)	111 (10%)	26 (11%)	2 (5%)	4 (6%)	0
Nicotine replacement		10 (<1%)	0	3 (1%)	5 (<1%)	2 (1%)	0	0	0
Not known		35 (2%)	3 (2%)	6 (1%)	13 (1%)	0	1 (2%)	3 (5%)	9 (60%)
**Diabetes**									
Yes		54 (3%)	4 (2%)	21 (5%)	17 (2%)	9 (4%)	0	3 (5%)	0
No		2013 (95%)	174 (96%)	408 (93%)	1099 (97%)	232 (95%)	41 (98%)	61 (95%)	8 (53%)
Not known		41 (2%)	3 (2%)	11 (2%)	17 (2%)	2 (1%)	1 (2%)	0	7 (47%)
**Previous radiotherapy to ipsilateral breast**									
Yes		139 (7%)	14 (8%)	26 (6%)	76 (7%)	17 (7%)	2 (5%)	5 (8%)	1 (7%)
No		1953 (95%)	167 (92%)	413 (94%)	1052 (93%)	223 (92%)	40 (95%)	59 (92%)	7 (47%)
Not known		16 (2%)	0	1 (<1%)	5 (<1%)	3 (1%)	0	0	7 (47%)
**Neoadjuvant chemotherapy**									
Yes		226 (11%)	28 (15%)	57 (13%)	111 (10%)	19 (8%)	1 (2%)	8 (13%)	2 (13%)
No		1853 (88%)	150 (83%)	381 (87%)	1006 (89%)	221 (91%)	41 (98%)	56 (88%)	7 (47%)
Not known		29 (1%)	3 (2%)	2 (<1%)	16 (1%)	3 (1%)	0	0	6 (40%)
**Neoadjuvant endocrine therapy**									
Yes		78 (4%)	11 (6%)	21 (5%)	37 (3%)	8 (3%)	1 (2%)	2 (3%)	1 (7%)
No		1999 (95%)	167 (92%)	415 (94%)	1079 (95%)	233 (96%)	41 (98%)	61 (95%)	9 (60%)
Not known		31 (1%)	3 (2%)	4 (1%)	17 (2%)	2 (1%)	0	1 (2%)	5 (33%)
**Previous surgery to ipsilateral breast**									
Yes		654 (31%)	61 (34%)	113 (26%)	371 (33%)	80 (33%)	8 (19%)	23 (36%)	4 (27%)
No		1434 (68%)	119 (66%)	322 (75%)	755 (67%)	162 (67%)	34 (81%)	41 (64%)	5 (33%)
Not known		20 (1%)	1 (1%)	5 (1%)	7 (<1%)	1 (<1%)	0	0	6 (40%)
**Type of previous surgery(s) received**									
Wide local excision		375 (57%)	29 (48%)	60 (53%)	218 (59%)	49 (61%)	3 (38%)	16 (70%)	3 (75%)
	With previous radiotherapy	108 (29%)	8 (28%)	17 (28%)	62 (28%)	15 (31%)	0	5 (31%)	1 (33%)
	Without previous radiotherapy	265 (71%)	21 (72%)	43 (72%)	155 (71%)	33 (69%)	3 (100%)	11 (69%)	2 (67%)
	Not known	2 (<1%)	0	0	1 (<1%)	1 (2%)	0	0	0
Sentinel node biopsy		299 (46%)	27 (44%)	48 (42%)	178 (48%)	31 (39%)	6 (75%)	10 (43%)	1 (25%)
Augmentation		49 (7%)	3 (5%)	5 (4%)	32 (9%)	6 (8%)	1 (13%)	2 (9%)	0
Reduction		28 (4%)	1 (2%)	5 (4%)	13 (4%)	6 (8%)	2 (25%)	3 (13%)	0
Other		116 (18%)	22 (36%)	19 (17%)	63 (17%)	14 (18%)	0	2 (9%)	0

Data are n (%) or median (IQR; range). Seven (<1%) of 2108 patients had missing data for all reported previous or neoadjuvant treatments.

* For patients with two implant-based reconstructions in which technique differed by breast (n=10), these patients are summarised in both applicable columns, depending on the method used, and once in the total column. Variations of approaches per breast within patient were biological mesh and dermal sling (n=3), biological mesh and synthetic mesh (n=1), dermal sling and submuscular or subfascial (n=4), other and submuscular or subfascial (n=1), and other and synthetic mesh (n=1).

† No mesh. 1261 (60%) patients reported no previous or neoadjuvant treatments.

‡ Could include a combination of techniques that could include or not include mesh.

**Table 2:** Operative details, by type of implant-based reconstruction

	All patients*(N=2108)	**Submuscular or subfascial**†**(n=181)**	**Dermal sling (n=440)**	**Biological mesh (n=1133)**	**Synthetic mesh (n=243)**	**Pre-pectoral (n=42)**	**Other (n=64)**	**Not known (n=15)**
**ASA grade**								
1	1246 (59%)	89 (49%)	206 (47%)	736 (65%)	155 (64%)	25 (60%)	36 (56%)	5 (33%)
2	783 (37%)	82 (45%)	213 (48%)	371 (33%)	80 (33%)	15 (36%)	25 (39%)	1 (7%)
3	57 (3%)	8 (4%)	15 (3%)	22 (2%)	7 (3%)	2 (5%)	2 (3%)	1 (7%)
4	0	0	0	0	0	0	0	0
Not known	22 (1%)	2 (1%)	6 (1%)	4 (<1%)	1 (<1%)	0	1 (2%)	8 (53%)
**Laterality**								
Bilateral	547 (26%)	141 (78%)	299 (68%)	866 (76%)	174 (72%)	21 (50%)	45 (70%)	15 (100%)
Unilateral	1561 (74%)	40 (22%)	141 (32%)	267 (24%)	69 (28%)	21 (50%)	19 (30%)	0
**Indication**‡§								
Malignancy only	1505 (71%)	137 (76%)	284 (65%)	835 (74%)	173 (71%)	20 (48%)	43 (67%)	13 (87%)
Risk reduction only	411 (19%)	27 (15%)	94 (21%)	209 (18%)	48 (20%)	18 (43%)	17 (27%)	0
Malignancy and contralateral risk-reducing surgery	188 (9%)	17 (9%)	62 (14%)	88 (8%)	22 (9%)	4 (10%)	3 (5%)	0
Not known	4 (<1%)	0	0	1 (<1%)	0	0	1 (2%)	2 (13%)
**Planned procedure**‡¶								
Single-stage reconstruction	1638 (78%)	77 (43%)	301 (68%)	969 (86%)	200 (82%)	36 (86%)	52 (81%)	7 (47%)
Two-stage reconstruction	453 (21%)	100 (55%)	134 (30%)	159 (14%)	42 (17%)	6 (14%)	12 (19%)	4 (27%)
Different approach per breast	12 (1%)	4 (2%)	5 (1%)	4 (<1%)	1 (<1%)	0	0	0
Not known	5 (<1%)	0	0	1 (<1%)	0	0	0	4 (27%)
**Type of mastectomy**‡‖**								
Skin-sparing	1161 (55%)	117 (65%)	96 (22%)	756 (67%)	149 (61%)	15 (36%)	24 (38%)	5 (33%)
Skin and nipple-sparing	486 (23%)	41 (23%)	21 (5%)	308 (27%)	81 (33%)	22 (52%)	15 (23%)	1 (7%)
Reduction pattern	398 (19%)	7 (4%)	308 (70%)	51 (5%)	6 (2%)	5 (12%)	22 (34%)	0
Other	18 (1%)	9 (5%)	3 (1%)	3 (<1%)	1 (<1%)	0	2 (3%)	0
Different type per breast	32 (2%)§	7 (4%)	12 (3%)	13 (1%)	4 (2%)	0	1 (2%)	0
Not known	13 (1%)	0	0	2 (<1%)	2 (1%)	0	0	9 (60%)
**Incision**††								
Peri-areolar	127 (6%)	10 (6%)	3 (1%)	90 (8%)	16 (7%)	3 (7%)	5 (8%)	0
Lateral	159 (8%)	13 (7%)	2 (<1%)	108 (10%)	29 (12%)	5 (12%)	2 (3%)	0
Inframammary	230 (11%)	20 (11%)	4 (1%)	149 (13%)	40 (16%)	13 (31%)	5 (8%)	1 (7%)
Elliptical removing NAC	936 (44%)	106 (59%)	13 (3%)	665 (59%)	123 (51%)	11 (26%)	17 (26%)	2 (13%)
Wise pattern	541 (26%)	11 (6%)	403 (92%)	77 (7%)	13 (5%)	7 (17%)	31 (48%)	1 (7%)
Other	78 (4%)	16 (9%)	6 (1%)	36 (3%)	16 (7%)	2 (5%)	3 (5%)	0
Different approaches per breast	19 (1%)¶	5 (3%)	6 (1%)	7 (1%)	3 (1%)	1 (2%)	1 (2%)	0
Not known	18 (1%)	0	3 (1%)	1 (<1%)	3 (1%)	0	0	11 (73%)
**Mastectomy weight, grams**‡††								
Median (IQR; range)	390 (260–583; 39–2300)	321 (208–456; 68–2300)	665 (492–915; 53–2260)	327 (229–476; 39–1480)	383 (264–540; 83–2200)	310 (230–527; 75–1544)	544 (332–790; 166–1505)	280 (280–280) (280–280)
Not known	90 (4%)	17 (9%)	13 (3%)	31 (3%)	9 (4%)	2 (5%)	5 (8%)	14 (93%)
**Prosthesis used**‡§§								
Fixed-volume implant	1235 (59%)	50 (28%)	210 (48%)	737 (65%)	166 (68%)	36 (86%)	39 (61%)	2 (13%)
Combined expander or implant	466 (22%)**	50 (28%)	102 (23%)	250 (22%)	46 (19%)	1 (2%)	18 (28%)	1 (7%)
Expander	383 (18%)	80 (44%)	125 (28%)	140 (12%)	29 (12%)	4 (10%)	7 (11%)	0
Different approaches per breast	4 (<1%)	0	0	4 (<1%)	1 (<1%)	0	0	0
Not known	20 (1%)	1 (<1%)	3 (1%)	2 (<1%)	1 (<1%)	1 (2%)	0	12 (80%)
**Axillary surgery**‡¶¶								
None	585 (28%)	46 (25%)	121 (28%)	315 (28%)	67 (28%)	20 (48%)	21 (33%)	0
SNB	881 (42%)	64 (35%)	177 (40%)	495 (44%)	106 (44%)	11 (26%)	23 (36%)	5 (33%)
Axillary sample	23 (1%)	5 (3%)	1 (<1%)	13 (1%)	3 (1%)	0	0	1 (7%)
Axillary clearance	226 (11%)	29 (16%)	48 (11%)	112 (10%)	24 (10%)	4 (10%)	9 (14%)	0
SNB and ANC	32 (2%)	3 (2%)	9 (2%)	15 (1%)	4 (2%)	0	1 (2%)	0
Previous axillary staging	164 (8%)	16 (9%)	23 (5%)	99 (9%)	16 (7%)	3 (7%)	7 (11%)	0
Different approach per breast	186 (9%)	18 (10%)	60 (14%)	83 (7%)	23 (9%)	4 (10%)	3 (5%)	0
Not known (%)	11 (<1%)	0	1 (<1%)	1 (<1%)	0	0	0	9 (60%)
**Operative time, min**								
Median (IQR; range)	180 (147–210; 60–570)	160 (130–195; 60–380)	186 (150–240; 62–530)	180 (150–210; 69–570)	165 (135–187; 70–410)	180 (147–240; 68–380)	180 (150–246; 60–480)	149 (118–180; 118–180)
Not known	195 (9%)	24 (13%)	37 (8%)	97 (9%)	4 (2%)	17 (40%)	3 (5%)	13 (87%)

Data are n (%), unless stated otherwise. ASA=American Society of Anesthesiologists. NAC=neoadjuvant chemotherapy. SNB=sentinel node biopsy. ANC=axillary node clearance.

* For patients with two implant-based reconstructions in which method differed by breast (n=10), these patients are summarised in both applicable columns, depending on the method used, and once in the total column. Variations of approaches per breast within patient were biological mesh and dermal sling (n=3), biological mesh and synthetic mesh (n=1), dermal sling and submuscular or subfascial (n=4), other and submuscular or subfascial (n=1), other and synthetic mesh (n=1).

† No mesh.

‡ Variable collected on a per-breast basis; for patients with two implant-based reconstructions, with data for one breast and not the other, that patient is classified according to the non-missing data as indicated in the applicable footnote.

§ Six patients classified according to non-missing data.

¶ One patient classified according to non-missing data.

‖ Five patients classified according to non-missing data.

** Combinations of type of mastectomy within patients with two implant-based reconstructions with different types per breast were skin-sparing mastectomy and skin and nipple-preserving mastectomy (n=15), skin-sparing mastectomy and reduction (Wise) pattern (n=7), skin-sparing mastectomy and other (n=3), skin and nipple preserving and reduction (Wise) pattern (n=2), skin and nipple preserving and other (n=4), reduction (Wise) pattern and other (n=1).

†† Six patients classified according to non-missing data. Combinations of location of incision within patients with two implant-based reconstructions with different types per breast were elliptical removing NAC and inframammary (n=1), elliptical removing NAC and lateral (n=4), elliptical removing NAC and other (n=3), elliptical removing NAC and peri-areolar (nipple preserving; n=3), elliptical removing NAC and Wise-pattern (n=1), inframammary and lateral (n=1), inframammary and other (n=2), inframammary and peri-areolar (nipple preserving; n=1), lateral and peri-areolar (nipple preserving; n=1), other and Wise pattern (n=2).

†† 19 patients classified according to non-missing data. When two patients had two implant-based reconstructions with mastectomy weight data for both breasts (n=511), the average weight is given. The difference in weight between breasts varied from 0 g to 463 g (median 40 g).

§§ Seven patients classified according to non-missing data. Combinations of breast prosthesis used within patients with two implant-based reconstructions with different types per breast were combined implant (Beckers) and fixed-volume implant (n=1), and fixed-volume implant and temporary expander (n=3).

¶¶ Four patients classified according to non-missing data. Combinations of axillary surgery within patients with two implant-based reconstructions with different surgeries per breast were axillary clearance and none (n=41), axillary clearance and previous staging axillary surgery (n=1), axillary clearance and sentinel node biopsy (n=10), axillary sample and none (n=6), none and previous staging axillary surgery (n=32), none and sentinel node biopsy and immediate clearance (n=2), none and sentinel node biopsy (n=46), and sentinel node biopsy and previous staging axillary surgery (n=1).

A one-stage reconstruction was planned in 1650 (78%) patients, and 1239 (59%) had a definitive fixed-volume implant placed at the time of their surgery. Two-stage reconstruction with tissue expanders or expandable implants was more common in patients having traditional submuscular procedures without mesh and those having dermal sling reconstruction than in those having other procedure types (table 2). Skin and nipple-sparing reconstruction was done in nearly a quarter of cases (table 2).

Approximately a third of the 1693 patients having mastectomy and immediate implant-based breast reconstruction for malignancy were recommended chemotherapy or radiotherapy after their reconstruction (table 3). This recommendation did not appear to be related to the type of procedure performed, although more patients having standard subpectoral reconstruction without mesh were recommended for radiotherapy than were patients having other procedure types (table 3).

**Table 3:** Postoperative data for patients having mastectomy for oncological indications

		All patients*(N=1693)	**Submuscular or subfascial**†**(n=154)**	**Dermal sling (n=340)**	**Biological mesh (n=922)**	**Synthetic mesh (n=194)**	**Pre-pectoral (n=24)**	**Other (n=46)**	**Not known (n=13)**
**Laterality**									
Unilateral malignancy		1633 (96%)	147 (95%)	329 (97%)	896 (97%)	184 (95%)	20 (83%)	44 (96%)	13 (100%)
Bilateral malignancy		60 (4%)	7 (5%)	11 (3%)	26 (3%)	10 (5%)	4 (17%)	2 (4%)	0
**Invasive status**									
Invasive		1223 (72%)	113 (73%)	244 (72%)	656 (71%)	144 (74%)	22 (92%)	34 (74%)	10 (77%)
Ductal carcinoma in situ		363 (21%)	25 (16%)	76 (22%)	214 (23%)	39 (20%)	1 (4%)	7 (15%)	1 (8%)
Different status per breast		11 (1%)	1 (1%)	1 (<1%)	8 (1%)	1 (1%)	0	0	0
Not known		96 (6%)	15 (10%)	19 (6%)	44 (5%)	10 (5%)	1 (4%)	5 (11%)	2 (15%)
**Grade**									
Low grade or well differentiated		131 (8%)	7 (5%)	20 (6%)	81 (9%)	15 (8%)	2 (8%)	3 (7%)	3 (23%)
Intermediate grade or moderately differentiated		653 (39%)	69 (45%)	137 (40%)	341 (37%)	75 (39%)	11 (46%)	15 (33%)	5 (38%)
High grade or poorly differentiated		418 (25%)	35 (23%)	80 (24%)	230 (25%)	50 (26%)	6 (25%)	15 (33%)	2 (15%)
Different per breast		14 (1%)	0	4 (1%)	5 (<1%)	1 (1%)	3 (13%)	1 (2%)	0
Not known		477 (28%)	43 (28%)	99 (29%)	265 (29%)	53 (27%)	2 (8%)	12 (26%)	3 (23%)
**Size of lesion, mm**									
Median (IQR; range)‡		21 (12–35; 0–750)	20 (16–50; 0–114)	23 (12–39; 0–750)	20 (12–31; 0–125)	28 (14–42; 0–120)	20 (14–36; 3–80)	20 (13–36; 0–150)	14 (7–19; 0–65)
Not known		480 (28%)	43 (28%)	98 (29%)	265 (29%)	54 (28%)	2 (8%)	15 (33%)	3 (23%)
**Number of involved nodes**									
Number (IQR; range)		0 (0–1; 0–54)§	0 (0–1; 0–22)	0 (0–1; 0–28)	0 (0–0; 0–32)	0 (0–1; 0–54)	0 (0–1; 0–5)	0 (0–1; 0–25)	0 (0–1; 0–0)
Not known		113 (7%)	15 (10%)	21 (6%)	53 (6%)	12 (6%)	1 (4%)	8 (17%)	3 (23%)
**Planned ANC for node-positive patients**									
Node-positive		425 (25%)	47 (31%)	91 (27%)	213 (23%)	59 (30%)	6 (25%)	10 (22%)	0
Yes planned ANC		193 (48%)¶	28 (62%)	34 (37%)	99 (50%)	29 (52%)	1 (17%)	2 (25%)	0
No planned ANC		206 (52%)¶	17 (38%)	53 (58%)	98 (50%)	27 (48%)	5 (83%)	6 (75%)	0
Not known		26 (6%)	2 (4%)	4 (4%)	16 (8%)	3 (5%)	0	2 (20%)	0
**Multidisciplinary team treatment recommendation**									
Adjuvant chemotherapy									
	Yes	562 (33%)	65 (42%)	103 (30%)	299 (32%)	71 (37%)	10 (42%)	13 (28%)	1 (8%)
	No	1002 (59%)	73 (47%)	212 (62%)	561 (61%)	112 (58%)	13 (54%)	26 (57%)	5 (38%)
	Not known	129 (8%)	16 (10%)	25 (7%)	62 (7%)	11 (6%)	1 (4%)	7 (15%)	7 (54%)
Adjuvant radiotherapy									
	Yes	495 (29%)	71 (46%)	101 (30%)	246 (27%)	62 (32%)	3 (13%)	11 (24%)	1 (8%)
	No	1055 (62%)	63 (41%)	210 (62%)	613 (66%)	118 (61%)	18 (75%)	28 (61%)	5 (38%)
	One breast only‖	17 (1%)	2 (1%)	4 (1%)	6 (1%)	3 (2%)	2 (8%)	0	0
	Not known	126 (7%)	18 (12%)	25 (7%)	57 (6%)	11 (6%)	1 (4%)	7 (15%)	7 (54%)
Adjuvant endocrine therapy									
	Yes	1081 (64%)	95 (62%)	209 (61%)	597 (65%)	126 (65%)	19 (79%)	29 (63%)	6 (46%)
	No	491 (29%)	40 (26%)	106 (31%)	275 (30%)	56 (29%)	4 (17%)	10 (22%)	0
	Not known	121 (7%)	19 (12%)	25 (7%)	50 (5%)	12 (6%)	1 (4%)	7 (15%)	7 (54%)

Data are n (%), unless otherwise stated. ANC=axillary node clearance.

* Includes data for 13 patients for whom mode of lower pole coverage was missing, who are not counted in the applicable type column.

† No mesh.

‡ When two patients have two malignant breasts with lesion size data for both breasts (n=38), the average lesion size is given. Size of lesions between breasts within these patients varied from 0 mm to 70 mm (median 10).

§ When two patients have two malignant breasts and there were data about the number of lymph nodes involved for both breasts (n=53), the average number of nodes is given. The number of lymph nodes between breasts varied from 0 to 15 (median 0).

¶ When two patients had two malignant breasts with planned axillary clearance data available for one breast, patients are classified according to the breast for which there are data (n=12 yes, n=9 no).

‖ When both breasts were operated for cancer but only one side needed radiotherapy.

Of the 2108 patients recruited, 2081 (99%) were followed up to 3 months (median follow-up 3 months, IQR 3–3). The clinical outcomes of interest are summarised in table 4 overall, and split by type of immediate implant-based breast reconstruction procedure. NMBRA data and quality standards for breast reconstruction^23^ are also included for comparison. Implant loss was experienced by 182 (9%) of 2081 patients followed up at 3 months, which is equivalent to the NMBRA published data (9%) but higher than National Quality Standards (<5%). 522 (25%) patients had a postoperative infection requiring treatment, and 372 (18%) patients were re-admitted for a complication after their reconstruction within 3 months. Both of these findings are consistent with results from the NMBRA^23^ and greater than the National Quality Standards (table 4). However, the proportion of patients requiring reoperation was greater than that in the NMBRA, with 370 (18%) patients in this cohort requiring further surgery for complications after their reconstruction procedure (table 4).

**Table 4:** 3-month outcomes after implant-based breast reconstruction, by procedure type, compared with outcomes in NMBRA and UK National Quality Criteria for Breast Reconstruction

	**All patients in iBRA with 3-month follow-up (n=2081)**	**Submuscular or fascial (n=180)**	**Dermal sling (n=436)**	**Biological mesh (n=1121)**	**Synthetic mesh (n=236)**	**Pre-pectoral (n=42)**	**Other (n=63)**	**Not known (n=11)**	**NMBRA outcomes at 3 months**	National Quality Criteria for Breast Reconstruction*
Reoperation	370; 18% (16–20)	30; 17% (12–23)	79; 18% (15–22)	193; 17% (15–20)	48; 20% (15–26)	9; 21% (10–37]	9; 14% (7–25)	2	5%	<5%
Re-admission	372; 18% (16–20)	31; 17% (12–24)	85; 19% (16–24)	185; 17% (14–19)	49; 21% (16–27)	10; 24% (12–40)	10; 16% (8–27)	2	16%	<5%
Infection	522; 25%, (23–27)	39; 22% (16–28)	138; 32% (27–36)	251; 22% (20–25)	61; 26% (20–32)	11; 26% (14–42)	19; 30% (19–43)	3	25%	<10%†
Implant loss	182; 9% (8–10)	17; 9% (6–15)	47; 11% (8–14)	90; 8% (7–10)	24; 10% (7–15)	3; 7% (2–20)	2; 3% (0–11)	1	9%	<5%

Data are n; % (95% CI), n, or %. NMBRA=National Mastectomy and Breast Reconstruction Audit.

* Oncoplastic Breast Reconstruction—Guidelines for Best Practice.

† Acellular dermal matrix-assisted breast reconstruction procedures: joint guidelines from the Association of Breast Surgery and the British Association of Plastic, Reconstructive and Aesthetic Surgeons. There were 2108 patients with implant-based reconstruction, of whom 2081 (99%) were included in the outcome analysis: complete outcome data (event data for all four key outcomes) are available for 2078 patients, who have been included in the analysis 27 (1%) patients have no outcome data and were excluded from the analysis. Partial outcome data (event data for three of four outcomes) are available for three patients, who were included in the analysis and who were assumed to not have had the event for the fourth missing outcome.

Using exploratory multivariable logistic regression, we identified an apparent association between body-mass index and smoking with all four clinical outcomes (table 5). This analysis also identified an apparent association between infection and previous radiotherapy, and between reoperation and operative time. Age, neoadjuvant chemotherapy, bilateral surgery, indication for surgery, nipple-sparing procedures, insertion of a definitive fixed-volume implant, and type of reconstruction performed were not significant risk factors for any of the key safety outcomes (table 5). Details of the number of events for each risk factor are shown in the appendix (p 8).

**Table 5:** Logistic regression of risk factors for key outcomes

		**Implant loss (n=1722)**	**Infection (n=1722)**	**Re-admission (n=1722)**	**Reoperation (n=1722)**
Age, years*		1·00 (0·98–1·02); p=0·87	1·01 (1·00–1·02); p=0·27	1·00 (0·99–1·01); p= 0·77	0·99 (0·98–1·01); p=0·35
Body-mass index (kg/m^2^)†		1·07 (1·03–1·11); p=0·0002	1·07 (1·04–1·10); p<0·0001	1·05 (1·03–1·08); p=0·0001	1·04 (1·01–1·07); p=0·0032
Operative time, min		1·00 (1·00–1·01); p=0·049	1·00 (1·00–1·00); p=0·073	1·00 (1·00–1·00); p=0·049	1·00 (1·00–1·01); p=0·013
Smoking					
	No	1 (ref)	1 (ref)	1 (ref)	1 (ref)
	Yes	1·92 (1·19–3·09); p=0·0074	1·53 (1·09–2·17); p=0·015	1·92 (1·33–2·77); p=0·0005	1·87 (1·30–2·70); p=0·0008
Previous radiotherapy to ipsilateral breast					
	No	1 (ref)	1 (ref)	1 (ref)	1 (ref)
	Yes	1·35 (0·70–2·60); p=0·37	1·72 (1·12–2·62); p=0·013	1·15 (0·69–1·91); p=0·59	1·24 (0·75–2·03); p=0·41
Neoadjuvant chemotherapy					
	No	1 (ref)	1 (ref)	1 (ref)	1 (ref)
	Yes	0·64 (0·33–1·21); p=0·17	0·72 (0·48–1·08); p=0·11	0·82 (0·53–1·28); p=0·38	0·73 (0·47–1·15); p=0·18
Bilateral surgery					
	No	1 (ref)	1 (ref)	1 (ref)	1 (ref)
	Yes	1·72 (0·85–3·47); p=0·13	1·27 (0·81–1·97); p=0·30	1·24 (0·76–2·03); p=0·39	1·15 (0·70–1·90); p=0·58
Nipple-sparing mastectomy					
	No	1 (ref)	1 (ref)	1 (ref)	1 (ref)
	Yes	1·24 (0·80–1·92); p=0·33	1·09 (0·82–1·46); p=0·55	1·04 (0·75–1·44); p=0·81	1·20 (0·88–1·64); p=0·25
Risk-reducing surgery					
	No	1 (ref)	1 (ref)	1 (ref)	1 (ref)
	Yes	0·87 (0·37–2·06); p=0·75	0·87 (0·48–1·56); p=0·64	1·13 (0·59–2·14); p=0·71	1·28 (0·68–2·41); p=0·45
Therapeutic mastectomy					
	No	1 (ref)	1 (ref)	1 (ref)	1 (ref)
	Yes	1·36 (0·69–2·69); p=0·38	0·80 (0·49–1·29); p=0·36	0·92 (0·55–1·54); p=0·74	1·11 (0·67–1·84); p=0·68
Fixed-volume implant					
	No	1 (ref)	1 (ref)	1 (ref)	1 (ref)
	Yes	0·87 (0·60–1·26); p=0·46	0·92 (0·72–1·16); p=0·46	0·86 (0·66–1·13); p=0·27	0·90 (0·69–1·18); p=0·45
Type of IBBR					
	Biological mesh	1 (ref)	1 (ref)	1 (ref)	1 (ref)
	Dermal sling	0·85 (0·52–1·38); p=0·50	1·21 (0·89–1·64); p=0·22	0·91 (0·64–1·30); p=0·60	0·85 (0·59–1·22); p=0·38
	Other	0·17 (0·02–1·25); p=0·082	1·34 (0·73–2·46); p=0·34	0·82 (0·39–1·74); p=0·60	0·80 (0·38–1·70); p=0·56
	Pre-pectoral	0·91 (0·20–4·04); p=0·90	1·02 (0·39–2·66); p=0·96	1·92 (0·76–4·82); p=0·17	1·37 (0·52–3·60); p=0·52
	Submuscular or fascial	1·06 (0·55–2·08); p=0·86	0·89 (0·56–1·41); p=0·63	1·03 (0·63–1·70); p=0·90	1·00 (0·61–1·63); p=0·98
	Synthetic mesh	1·12 (0·66–1·90); p=0·68	1·13 (0·79–1·61); p=0·50	1·20 (0·81–1·78); p=0·37	1·09 (0·74–1·62); p=0·66

Data are odds ratio (95% CI); p value. IBBR=immediate implant-based breast reconstruction.

* Increase in odds for each additional year.

† Increase in odds for each additional body-mass index unit.

## Discussion

This national, multicentre, prospective cohort study of 2108 patients having immediate implant-based breast reconstruction in 81 centres across the UK shows that the short-term clinical outcomes of immediate implant-based breast reconstruction fall far short of the published aspirational quality standards for immediate breast reconstruction,^23^ and have not improved in the 10 years since the NMBRA.^24^ Despite recently published evidence showing increased frequency of complications in mesh-assisted immediate implant-based breast reconstruction,^11^ there was no association between type of mesh and short-term safety outcomes in exploratory regression analyses of this large, non-randomised study. The optimum technique for immediate implant-based breast reconstruction is therefore unknown and more comparative data are needed. To truly answer this important question, a large-scale, pragmatic, randomised controlled trial will be required to identify the most clinically and cost-effective approach to immediate implant-based breast reconstruction, and provide information to inform clinical and health policy decisions.

Despite insufficient high-quality evidence, mesh-assisted, single-stage, direct-to-implant reconstruction using fixed-volume or adjustable implants has become the most widely used procedure in the UK,^22^ with less than 10% of patients undergoing traditional two-stage expander-implant procedures. This widespread adoption of mesh suggests that a randomised controlled trial attempting to compare single-stage, direct-to-implant reconstruction with mesh and the standard two-stage techniques would be difficult because of surgeon preference. However, our study shows little difference in the short-term clinical outcomes of biological and synthetic mesh-assisted immediate implant-based breast reconstruction, and has highlighted the large number of products in current use. Pre-pectoral reconstruction was done only in a small number of patients in this study, although it is gaining popularity.^7^ Despite the challenges associated with a randomised clinical trial in breast reconstruction, methods have been developed and successfully used to overcome these issues and support surgeons to recruit patients into trials of very different types of procedures, in which patient and surgeon preferences might be strong.^27^

Although the type of reconstructive technique used does not appear to affect short-term safety outcomes, patient factors such as increased BMI and current smoking seemed to be associated with increased risks of implant loss, infection, re-admission, and reoperation. Additionally, these results indicate that previous radiotherapy might be associated with a modest increase in the odds of developing a postoperative infection. Although this analysis was exploratory in nature, these results highlight the importance of careful patient selection and providing patients at high risk with accurate information about the likelihood of postoperative complications, to allow them to make better informed decisions. Operative time was another factor potentially associated with increased risk of some major complications, particularly reoperation. However, the effects of measures to reduce operating time (eg, dual-surgeon operating for bilateral cancer, or doing a contralateral risk-reducing mastectomy in patients with unilateral malignancy as a delayed procedure) could potentially reduce complications, but this remains to be determined.

The proportion of patients identified in this study who experienced implant loss, re-admission, and infection remain unchanged since the 2008–09 NMBRA, whereas the proportion of patients requiring re-operation has more than tripled.^24^ The reasons for this increase are complex. The numbers of immediate implant-based breast reconstruction have increased substantially since 2008–09,^5^, ^22^ but there is no evidence to suggest that the indications for implant-based surgery have changed, since the proportions of patients who smoked, had diabetes, had a high BMI, or were American Society of Anesthesiologists grades III/IV in our study are largely consistent with the NMBRA cohort.^24^ Increased numbers of re-operations could reflect more aggressive management of complications when mesh is used, but the use of mesh does not seem to translate into a reduced percentage of implant loss. Although the proportions of patients with implant loss and return to theatre in this study are much lower than those reported in a 2017 randomised trial,^11^, ^13^ they are much higher than those reported in other large prospective observational studies^14^, ^15^, ^28^ and summarised in recent systematic reviews.^8^, ^9^ This large, multicentre study is likely to reflect real-world outcomes of immediate implant-based breast reconstruction, and highlights the need for improvement in this area.

This national prospective study adds substantially to the evidence base in immediate implant-based breast reconstruction, but has several limitations. Firstly, it is a non-randomised study that will be subject to bias. Notably, patients having dermal sling reconstruction had higher BMIs than did those in the other groups, but other, subtler, differences might exist between patients having different procedures that were not considered in this study. Despite defining outcomes, including implant loss, a priori, practice changed during the study period. Of particular note was the introduction of implant salvage procedures, whereby fixed-volume implants that were infected or exposed were debrided, washed out, and replaced either with a new implant or a tissue expander. This outcome was not considered to be an implant loss in our study, although the initial implant was removed. Infection was another controversial topic. We considered any redness requiring treatment with antibiotics in this category. We acknowledge that this might have overestimated the occurrence of implant infection, but reported frequencies were consistent those in the NMBRA.^24^ Clear, unambiguous definitions of outcomes will therefore be needed for future studies. Smoking, BMI, operative time, and previous radiotherapy were identified as risk factors for complications in this study, and this potential association might be informative when designing subsequent randomised clinical trials as a basis for balancing randomisation. Although this study was not powered to establish prognostic factors, and the results should be confirmed in an external validation study, consistency between these results and those in other studies^29^, ^30^ support these findings. Finally, complications were only assessed until 3 months. This approach would not capture complications, such as infection, which developed while patients were receiving chemotherapy or problems developing as a result of adjuvant radiotherapy, for which a longer period of follow-up would be required.

The findings of this large, non-randomised study strongly support the need for a randomised controlled trial in immediate implant-based breast reconstruction, and potential trial designs could include biological versus synthetic mesh or pre-pectoral versus sub-pectoral implant placement. Before embarking on a full trial, a pilot randomised clinical trial is recommended, to establish whether recruitment is possible. Additionally, urgent work is also needed to improve outcomes for patients having immediate implant-based breast reconstruction in the UK. The proportion of patients experiencing implant loss and infection and those requiring re-operation and re-admission do not appear to have improved (ie, decreased) since NMBRA, and do not meet published quality standards. Reasons for this finding are unclear, but non-compliance with best practice guidelines might be a contributory factor^21^ and further investigation of variation by centre is planned. This study provides further evidence that increased BMI and smoking significantly increase the risk of complications after implant-based breast reconstruction. These factors are not immediately modifiable in the short-term, but neoadjuvant chemotherapy or endocrine therapy could be used as a strategy to provide patients with breast cancer with additional time to lose weight or stop smoking before surgery. Bilateral risk-reducing surgery, meanwhile, could be delayed until these risk factors had been addressed. An alternative solution would be to restrict the offer of immediate implant-based breast reconstruction to patients without risk factors. This approach might not be practical or ethical, and a more appropriate focus could be to develop effective strategies to help patients better understand the potential risks of surgery, to allow them to make fully informed decisions. Finally, reducing the observed variation might effectively improve outcomes, and this will be the focus of the UK Getting it Right First Time initiative. However, it is important that any standardisation of care reflects evidence-based best practice, and further exploratory analysis of the iBRA cohort will support this.

There remains the need for high-quality evidence from a randomised controlled trial to support the best practice of immediate implant-based breast reconstruction, and the equivalence of different techniques in this non-randomised study supports a future randomised clinical trial. The outcomes of immediate implant-based breast reconstruction in the UK are poor and surgeons need to commit to robust evaluation if outcomes for patients are to be improved.

For more on **REDCap** see http://www.projectredcap.org/

For more on the **HRA decision tool** see http://www.hradecisiontools.org.uk/research

For more on the **Getting it Right First Time initiative** see http://gettingitrightfirsttime.co.uk/surgical-specialty/breast-surgery
