# Supplementary material for: Short-term safety outcomes of mastectomy and immediate implant-based breast reconstruction with and without mesh (iBRA): a multicentre, prospective cohort study
Source: Lancet Oncol. 2019 Feb;20(2):254–66. doi: 10.1016/S1470-2045(18)30781-2 (PMC6358590; doi:10.1016/S1470-2045(18)30781-2)
Supplement: Supplementary appendix [file mmc1.pdf]

THE LANCET
Oncology

Supplementary appendix

This appendix formed part of the original submission and has been peer reviewed. We post it as supplied by the authors.

Supplement to: Potter S, Conroy EJ, Cutress RI, et al. Short-term safety outcomes of mastectomy and immediate implant-based breast reconstruction with and without mesh (iBRA): a multicentre, prospective cohort study. *Lancet Oncol* 2019; published online Jan 9. http://dx.doi.org/10.1016/S1470-2045(18)30781-2.

Web Appendix for Short-term safety outcomes of immediate implant-based breast reconstruction with and without mesh: The iBRA multicentre prospective cohort study

The iBRA Steering Group (in alphabetical order) comprises: N L P Barnes, J M Blazeby, O A Branford, E J Conroy, R I Cutress, M D Gardiner, C Holcombe, A Jain, K McEvoy, N Mills, S Mylvaganam, S Potter, J M Skillman, E M Teasdale, S Thrush, Z Tolkien, L J Whisker, P R Williamson

Local investigators (alphabetically by centre) and members of the Breast Reconstruction Research Collaborative are PUBMED citable collaborators in this study and were:

Airedale General Hospital: P Christopoulos, V Fung, C Murphy, L Caldon, H Fatayer, E Baker; **Altnagelvin Area Hospital:** R Johnston; **Ashford and St Peters NHS Trust**: R Newton, A Luangsomboon, B Swiech, A Robinson, M Runkel, **Barts Health NHS Trust:** D Zheng, F Tsang, L Johnson, A Peel, S Ledwidge, S Barker, J Hu, V Voynov, G Exarchos, N Jiwa; **Barnsley Hospital NHS Foundation Trust:** OS Olayinka, JR Dicks; **Basingstoke and North Hampshire Hospital:** V Kalles, K Harris; **Bedford Hospital NHS:** N Manoloudakis, H Charfare, F Conroy; **Belfast City Hospital:** G Dobson, S Sloan, G Irwin, L Darragh, S McIntosh, S Refsum, S Dawson; **Blackpool Teaching Hospital NHS Foundation Trust:** I Michalakis, D Debnath, N Geerthan, P Kiruparan; **Bradford Teaching Hospitals NHS Foundation Trust:** S Hignett, E Baker, C Tait, R Linforth, M Salab, K Rigby; **Brighton & Sussex University Hospital NHS Trust:** S Shaheed, F Ugolini, R Rathinaezhil, C Zammit, H Osman, A Chouhan; **Castle Hill Hospital, Hull:** A Wilkins, B Wooler, P Kneeshaw, T K Mahapatra, E Khalifa, K Grover, K Hodgkins, S Harrison, P McManus, E Mallidis, J Robinson, F Langlands; **Chesterfield Royal Hospital:** F Mazari, J Massey, I Azmy, C Hollywood; **Countess of Chester Hospital:** W Hamilton-Burke, H Lennon; C Harding-Mackean; **County Durham and Darlington NHS Trust:** T Collin, J Henton; **Craigavon Area Hospital:** G Irwin, P Mallon; **Diana Princess of Wales Hospital, Grimsby:** J Smith, T Masudi, S Joglekar; **Dorset County Hospital:** K Kennedy, T Graja, C Osborne, R. Sutaria, M Youssef, T Stringfellow; **Dunedin Hospital:** B Smith; **East Lancashire Teaching Hospitals**: A.Topps, M Amanita, S Gawne, J McNicholas, C Thomas, M Khanbhai, R Brindle, N Taheri, R Kuruvilla, M Saleh, F Bux, P Pugh, J Iddon; **East Surrey Hospital:** E J Turner, S Waheed; **Frimley Park Hospital:** D Egbeare, J Stevens, E Mallidis, R Daoud, I Karat, I Laidlaw, H Osman, K Kanesalingam, R Johnson, R Gurung; **Glenfield Hospital Leicester:** K Lambert, S Pilgrim, T Rattay, H Khout, D Appleton, B Vijaynagar, S Bains, M Kaushik, J Krupa; **Great Western Hospital, Swindon:** I Dash, A Chaudry, M Galea, N Coombs; **Homerton Hospital:** S Tayeh, S Darvesh, C Choy, L Parvanta; **Kettering General Hospital:** A Knight, M Wright, G Wilson, S Musa; **King George Hospital, Ilford:** S Saha, S Samlalsingh, A Ogedegbe; **Lancashire Teaching Hospitals – Chorley:** A Topps, N Bishop, G Boland. Z Saidan, B Murthy; **Leighton Hospital:** S Hignett, V Pope **; Lincoln County Hospital:** A Sami, A Giaramadze; **Luton and Dunstable University Hospital:** R James, E Stewart-Parker, K Kirkpatrick, D Ravichandran; **Milton Keynes NHS Trust:** A Taylor, K Chin; **Maidstone and Tumbridge Wells NHS Trust:** M Hashem, R Chalmers, H Devalia, K Cox, S Horn, M Dani, D Akolekar, C Chalmers; **Musgrove Park Hospital, Taunton:** S Potter, A Thorne, S Granger; J Gill, U Hassan, C Ives, T Walker, M Chana; **Nevill Hall Hospital:** V Lefemine, F Soliman; **New Cross Hospital, Wolverhampton:** T Sircar, F Salem, S Mylvaganam, P Matey, G Nagra, S Marla, R Vidya, N Nuru, I Adwan, E Fage, O Al-Jibury; **North Bristol NHS Trust:** S Potter, I Dash, S Cawthorn, J Cook, S Govindarajulu; **North Middlesex University Hospital:** T Gandamihardja; **Nottingham Breast Unit:** L Brock, M Akelund, C Otieno, A Halka, R D Macmillan, K Asgiersson, L Whisker, H Khout, T Rasheed, C Laban, E Gutteridge, S McCulley; **Oxford:** D Remoundos, PJ Roy; **Peterborough City Hospital:** E Popa, Mr Goh, G Shetty; **Poole General Hospital:** S Clark, A Evans; **Prince Phillip Hospital:** S Udayasankar, S Khawaja, Y Sharaiha; **QE2 Welwyn Garden City**: U Walsh, H Deol; **Royal Berkshire Hospital:** N Dunne, B Smith, A Hakim; **Royal Bolton Hospital:** A Volleamere, C Garnsey, C Wright; **Royal Bournemouth Hospital:** E Skene; C Laban, A Iskender, D Perry; **Royal Devon and Exeter NHS Foundation Trust:** D Egbeare, M Wiltsher, R Johnston, D Ferguson; S Olsen, R Tillett, M Youssef; **Royal Glamorgan Hospital:** S Datta, G Patel, C Steventon, R Foulkes, E Vaughan Williams, G Osborn, C Gateley; **Royal Hampshire County Hospital, Winchester:** N Chand, B Zeidan, D Rainsbury, S Laws. L Peiris, K Harris, V Kalles; **Royal Liverpool and Broadgreen University Hospitals:** A Hargreaves, J Henderson, T Kiernan, N Barnes, M Chandrashekar, A Tansley, C Holcombe, G Mitchell, R Little, S Bathla, M Pennick, E De Sousa, W Hamiton-Burke; **Royal Marsden Hospital:** R Di Micco, R O'Connell, V Voynov, A Parvaiz, A Conway, P Barry, G Gui, J Rusby, N Roche, K Ramsey, K Krupa, L Johnson, D Elfadl, F MacNeil, A Peppe, L Soldanova, I Hamo, P Harris, A Augusti, C Constantinou, A R Godden; **Royal Surrey County Hospital:** E Clayton, A Conway, T Irvine, P Partlett, F Pakzad; **Royal United Hospital Bath:** R Shah, I Dash, S Potter, J Mcintosh, R Sutton, N Laurence; **Royal Victoria Infirmary, Newcastle:** L Darragh, J O'Donoghue, S Nicholson, H Cain, N Collis, J Chatterjee, A Critchley, L Caldon; **St. James's University Hospital, Leeds:** B Kim, D Remoundos, J Massey, R Achuthan, C Fenn, F Mazari, C Navin, K Horgan, S McKenzie, P Turton, M Lansdown; **St George's Hospital:** S Tang, Mr Banerjee; **St Marys Hospital, Isle of Wight:** M Jobson; **Salisbury District Hospital:** S Masood, V Brown, G Murphy; **Sheffield Teaching Hospitals NHS Trust:** V Fung, N Dunne, L Wyld. S Kohlhardt, V Chandran, L Maraqa, L Caldon, M Reed, A Ismail; **Southampton:** S Robertson, R Cutress; **Torbay and South Devon NHS Foundation Trust:** C Ives, M Green; **University College Hospital, London:** L Johnson, R Carpenter, J Gattuso, J Franks; **University Hospitals Birmingham:** S Mylvaganam, R Warner, L MacLennan, J Dickson, R Waters, N Basu, S Thomas, S Tadiparthi, S Aggarwal; **University Hospital Coventry and Warwickshire:** A Carbone, J Skillman, T Challoner, S Parker, A Park, A Tomlins, H Khan, E Eltigani, M Kishore, R Nangalia, S Chambers, S Ayaani; **University Hospital Llandough:** C Thomas, R Foulkes, A Hussain, J Iddon, H Sweetland, E Davies, S Tate, S Goyal, A Ghattura, C Zabkiewicz; **University Hospitals North Manchester:** S Narayanan, S Soumian, D Archampong, E Erel; **University Hospitals North Staffordshire:** S Narayanan, S Soumian, V Voynov; **University Hospitals South Manchester:** J Henderson, N Barnes, R Shotton, R Johnson, A Gandhi, J Harvey, C Kirwan, J Murphy, G Byrne, D El Sharief, B Baker, R Chattopadhyay, S Chatterjee, R Irri, Mei Ju Hwang, K Williams; L Barr; **Warrington and Halton Hospitals:** N Sarfraz, P Thawdar; **Warwick Hospital:** M Dakka, H Tafazal, L Jones, D Enver, D Clarke, S Harries; **West Hertfordshire Hospital NHS Trust:** D Cocker, S Monib, L M Lai, S Thomson, K Chong, S Baldota, J Maalo; **Whiston Hospital:** N Barnes, T Kiernan, R Clifford, M Pennick, L Chagla, A Iqbal; **Wirral University Teaching Hospital NHS Foundation Trust:** R Vinayagam, J Lund, M Callaghan, S Poonawalla, K James; **Worcestershire Royal Hospital**: M Tan, R Athwal, M Mullan, S Thrush, R Bright-Thomas, J Taylor, H Tafazal, K McEvoy, M Ahmed; **Yeovil District Hospital:** N Dobner, C Osborne; **York NHS Foundation Trust:** J Piper, E Baker, R Nasr.

Supplementary table 1: Missing data within each risk factor for each key outcome

		Reoperation		Readmission		Infection		Implant loss	
	**Total**	**Yes**	**No**	**Yes**	**No**	**Yes**	**No**	**Yes**	**No**
**Number of patients**	2081	370	1711	372	1709	522	1559	182	1899
**Age**	10<br>0.5%	3<br>0.8%	7<br>0.4%	3<br>0.8%	7<br>0.4%	4<br>0.8%	6<br>0.4%	1<br>0.5%	9<br>0.5%
**Smoking**	30<br>1.4%	7<br>1.9%	23<br>1.3%	5<br>1.3%	25<br>1.5%	9<br>1.7%	21<br>1.3%	3<br>1.6%	27<br>1.4%
**BMI**	118<br>5.7%	26<br>7.0%	92<br>5.4%	27<br>7.3%	91<br>5.3%	22<br>4.2%	96<br>6.2%	11<br>6.0%	107<br>5.6%
**Previous radiotherapy**	13<br>0.6%	3<br>0.8%	10<br>0.6%	3<br>0.8%	10<br>0.6%	3<br>0.6%	10<br>0.6%	0<br>0.0%	13<br>0.7%
**Neo adjuvant chemo**	26<br>1.2%	8<br>2.2%	18<br>1.1%	7<br>1.9%	19<br>1.1%	8<br>1.5%	18<br>1.2%	4<br>2.2%	22<br>1.2%
**Length of operation**	189<br>9.1%	28<br>7.6%	161<br>9.4%	34<br>9.1%	155<br>9.1%	49<br>9.4%	140<br>9.0%	17<br>9.3%	172<br>9.1%
**Bilateral**	0<br>0.0%	0<br>0.0%	0<br>0.0%	0<br>0.0%	0<br>0.0%	0<br>0.0%	0<br>0.0%	0<br>0.0%	0<br>0.0%
**Nipple sparing**	10<br>0.5%	2<br>0.5%	8<br>0.5%	2<br>0.5%	8<br>0.5%	2<br>0.4%	8<br>0.5%	2<br>1.1%	8<br>0.4%
**Risk reducing vs. therapeutic**	4<br>0.2%	0<br>0.0%	4<br>0.2%	0<br>0.0%	4<br>0.2%	0<br>0.0%	4<br>0.3%	0<br>0.0%	4<br>0.2%
**Fixed vs. adjustable**	17<br>0.8%	4<br>1.1%	13<br>0.8%	4<br>1.1%	13<br>0.8%	4<br>0.8%	13<br>0.8%	4<br>2.2%	13<br>0.7%

Supplementary table 2: Unit specific recruitment and local collaborators in the iBRA Study

Hospital name	Local Collaborators	Date participant recruited		Total number recruited		Active recruitment months[1]		All recruitment months[2]	
		First	Last			Months actively recruiting	Monthly recruitment rate	Months actively recruiting	Monthly recruitment rate
**Overall**		**04/02/2014**	**30/06/2016**	**2108**		**28**	**75**	**28**	**75**
Royal Liverpool University Hospital	A Hargreaves<br>J Henderson*<br>T Kiernan<br>N Barnes<br>M Chandrashekar*<br>A Tansley*<br>C Holcombe*<br>G Mitchell*<br>R Little<br>S Bathla<br>M Pennick<br>E De Sousa*<br>W Hamilton-Burke	04/02/2014	22/06/2016	118	5.6%	28	4	28	4
University Hospitals South Manchester	J Henderson<br>N Barnes*<br>R Shotton<br>R Johnson*<br>A Gandhi*<br>J Harvey*<br>C Kirwan*<br>J Murphy*<br>G Byrne*<br>D El Sharief<br>B Baker<br>R Chattopadhyay<br>S Chatterjee<br>R Irri<br>Mei Ju Hwang<br>K Williams<br>L Barr*	12/01/2015	30/06/2016	115	5.5%	17	7	17	7
Nottingham Breast Unit	L Brock<br>M Akelund<br>C Otieno<br>A Halka<br>R D Macmillan*<br>K Asgiersson*<br>L Whisker*<br>H Khout*<br>T Rasheed*<br>C Laban<br>E Gutteridge*<br>S McCulley*	04/08/2014	27/06/2016	106	5.0%	22	5	22	5
Royal Marsden Hospital	R Di Micco<br>R O'Connell<br>V Voynov<br>A Parvaiz<br>A Conway<br>P Barry*<br>G Gui*<br>J Rusby*<br>N Roche*<br>K Ramsey*<br>K Krupa*<br>L Johnson<br>D Elfadl<br>F MacNeil*<br>A Peppe<br>L Soldanova<br>I Hamo<br>P Harris*<br>A Augusti<br>C Constantinou<br>A R Godden	20/05/2014	28/06/2016	101	4.8%	25	4	25	4
University Hospital Coventry and Warwickshire:	A Carbone<br>J Skillman*<br>T Challoner<br>S Parker*<br>A Park*<br>A Tomlins*<br>H Khan*<br>E Eltigani*<br>M Kishore*<br>R Nangalia<br>S Chambers<br>S Ayaani;	26/03/2014	22/02/2016	85	4.0%	22	4	27	3
New Cross Hospital, Wolverhampton	T Sircar*<br>F Salem<br>S Mylvaganam*<br>P Matey*<br>G Nagra<br>S Marla<br>R Vidya*<br>N Nuru<br>I Adwan<br>E Fage<br>O Al-Jibury	17/11/2014	24/06/2016	82	3.9%	19	4	19	4
Brighton & Sussex University Hospital NHS Trust	S Shaheed<br>F Ugolini<br>R Rathinaezhil<br>C Zammit*<br>H Osman*<br>A Chouhan	20/06/2014	09/06/2016	69	3.3%	23	3	24	3
Maidstone & Tumbridge Wells NHS Trust	M Hashem*<br>R Chalmers*<br>H Devalia*<br>K Cox*<br>S Horn*<br>M Dani<br>D Akolekar*<br>C Chalmers	11/07/2014	24/06/2016	64	3.0%	23	3	23	3
West Hertfordshire Hospital NHS Trust	D Cocker<br>S Monib<br>L M Lai*	04/02/2014	09/06/2016	62	2.9%	28	2	28	2

Hospital name	Local Collaborators	Date participant recruited		Total number recruited		Active recruitment months[1]		All recruitment months[2]	
		First	Last			Months actively recruiting	Monthly recruitment rate	Months actively recruiting	Monthly recruitment rate
**Overall**		**04/02/2014**	**30/06/2016**	**2108**		**28**	**75**	**28**	**75**
	S Thomson*<br>K Chong*<br>S Baldota<br>J Maalo								
Wirral University Teaching Hospital NHS Foundation Trust	R Vinayagam*<br>J Lund*<br>M Callaghan*<br>S Poonawalla*<br>K James*	05/09/2014	28/06/2016	61	2.9%	21	3	21	3
Blackpool Teaching Hospital NHS Foundation Trust	I Michalakis*<br>D Debnath*<br>N Geerthan<br>P Kiruparan*	09/01/2015	30/06/2016	60	2.9%	17	4	17	4
Sheffield Teaching Hospitals NHS Trust	V Fung<br>N Dunne<br>L Wyld*<br>S Kohlhardt*<br>V Chandran*<br>L Maraqa*<br>L Caldon<br>M Reed*<br>A Ismail*	25/11/2014	28/06/2016	54	2.6%	19	3	19	3
Belfast City Hospital	G Dobson<br>S Sloan*<br>G Irwin<br>L Darragh<br>S McIntosh*<br>S Refsum*<br>S Dawson;	10/06/2014	05/01/2016	53	2.5%	18	3	24	2
Worcestershire Royal Hospital	M Tan<br>R Athwal<br>M Mullan*<br>S Thrush*<br>R Bright-Thomas*<br>J Taylor*<br>H Tafazal<br>K McEvoy<br>M Ahmed	16/04/2014	30/06/2016	52	2.5%	26	2	26	2
St. James's University Hospital Leeds	B Kim<br>D Remoundos<br>J Massey<br>R Achuthan*<br>C Fenn*<br>F Mazari<br>C Navin*<br>K Horgan*<br>S McKenzie*<br>P Turton*<br>M Lansdown	16/07/2014	11/05/2016	49	2.3%	21	2	23	2
Airedale General Hospital	P Christopoulos<br>V Fung,<br>C Murphy*<br>L Caldon,<br>H Fatayer<br>E Baker	12/03/2014	18/05/2016	48	2.3%	26	2	27	2
Frimley Park Hospital	D Egbeare*<br>J Stevens<br>E Mallidis<br>R Daoud*<br>I Karat*<br>I Laidlaw*<br>H Osman<br>K Kanesalingam<br>R Johnson<br>R Gurung	02/09/2014	28/06/2016	46	2.2%	21	2	21	2
Barts Health NHS Trust	D Zheng<br>F Tsang<br>L Johnson<br>A Peel<br>S Ledwidge*<br>S Barker<br>J Hu*<br>V Voynov*<br>G Exarchos<br>N Jiwa;	10/07/2014	01/06/2016	44	2.1%	22	2	23	2
Royal Bolton Hospital	A Volleamere*<br>C Garnsey*<br>C Wright	07/07/2014	14/06/2016	43	2.0%	23	2	23	2
Glenfield Hospital Leicester	K Lambert<br>S Pilgrim*<br>T Rattay<br>H Khout*<br>D Appleton<br>B Vijaynagar<br>S Bains<br>M Kaushik<br>J Krupa	26/01/2015	28/06/2016	39	1.9%	17	2	17	2
Royal Victoria Infirmary, Newcastle	L Darragh<br>J O'Donoghue*<br>S Nicholson*<br>H Cain*<br>N Collis*<br>J Chatterjee*<br>A Critchley*<br>L Caldon	15/04/2015	23/05/2016	39	1.9%	13	3	14	3
Royal Devon and Exeter NHS Foundation Trust	D Egbeare<br>M Wiltsher<br>R Johnston<br>D Ferguson*<br>S Olsen*<br>R Tillett*<br>M Youssef	30/06/2014	25/04/2016	37	1.8%	21	2	24	2
University Hospitals Birmingham	S Mylvaganam<br>R Warner*<br>L MacLennan	11/09/2014	08/03/2016	35	1.7%	17	2	21	2

Hospital name	Local Collaborators	Date participant recruited		Total number recruited		Active recruitment months[1]		All recruitment months[2]	
		First	Last			Months actively recruiting	Monthly recruitment rate	Months actively recruiting	Monthly recruitment rate
**Overall**		**04/02/2014**	**30/06/2016**	**2108**		**28**	**75**	**28**	**75**
	J Dickson*<br>R Waters*<br>N Basu*<br>S Thomas*<br>S Tadiparthi*<br>S Aggarwal*								
Royal United Hospital Bath	R Shah<br>I Dash<br>S Potter<br>J Mcintosh*<br>R Sutton*<br>N Laurence*	25/04/2015	29/06/2016	34	1.6%	14	2	14	2
Bradford Teaching Hospitals NHS Foundation Trust	S Hignett*<br>E Baker<br>C Tait<br>R Linforth*<br>M Salab*<br>K Rigby*	02/09/2014	20/06/2016	34	1.6%	21	2	21	2
Homerton Hospital	S Tayeh<br>S Darvesh*<br>C Choy*<br>L Parvanta*	25/09/2014	30/06/2016	34	1.6%	21	2	21	2
Dorset County Hospital	K Kennedy<br>T Graja*<br>C Osborne*<br>R. Sutaria<br>M Youssef<br>T Stringfellow	22/07/2014	07/06/2016	28	1.3%	22	1	23	1
Royal Berkshire Hospital	N Dunne*<br>B Smith*<br>A Hakim	19/01/2015	03/05/2016	27	1.3%	15	2	17	2
Castle Hill Hospital Hull	A Wilkins<br>B Wooler<br>P Kneeshaw*<br>T K Mahapatra*<br>E Khalifa*<br>K Grover*<br>K Hodgkins<br>S Harrison<br>P McManus*<br>E Mallidis*<br>J Robinson<br>F Langlands	13/01/2015	22/06/2016	26	1.2%	17	2	17	2
Diana Princess of Wales Hospital, Grimsby	J Smith*<br>T Masudi<br>S Joglekar	16/06/2014	30/09/2015	23	1.1%	15	2	24	1
Royal Hampshire County Hospital, Winchester	N Chand<br>B Zeidan<br>D Rainsbury*<br>S Laws*<br>L Peiris*<br>K Harris*<br>V Kalles*	16/07/2014	21/06/2016	23	1.1%	23	1	23	1
Musgrove Park Hospital, Taunton	S Potter<br>A Thorne*<br>S Granger<br>J Gill*<br>U Hassan<br>C Ives<br>T Walker<br>M Chana	13/08/2014	01/06/2016	22	1.0%	21	1	22	1
East Lancashire Teaching Hospitals	A.Topps<br>M Amanita<br>S Gawne*<br>J McNicholas*<br>C Thomas<br>M Khanbhai<br>R Brindle<br>N Taheri<br>R Kuruvilla<br>M Saleh<br>F Bux<br>P Pugh<br>J Iddon	08/09/2014	07/10/2015	19	0.9%	12	2	21	1
Luton and Dunstable University Hospital	R James*<br>E Stewart-Parker*<br>K Kirkpatrick*<br>D Ravichandran*	04/02/2015	07/01/2016	19	0.9%	11	2	16	1
Milton Keynes NHS Trust	A Taylor*<br>K Chin*	21/10/2014	01/12/2015	19	0.9%	13	1	20	1
North Bristol NHS Trust	S Potter<br>I Dash<br>S Cawthorn*<br>J Cook<br>S Govindarajulu*	12/11/2014	15/09/2015	17	0.8%	10	2	19	1
University Hospitals North Manchester	S Narayanan*<br>S Soumian*<br>D Archampong<br>E Erel	27/01/2016	26/05/2016	16	0.8%	3	5	5	3
University Hospital Llandough	C Thomas<br>R Foulkes*<br>A Hussain<br>J Iddon*<br>H Sweetland*<br>E Davies*<br>S Tate<br>S Goyal<br>A Ghattura*<br>C Zabkiewicz	11/11/2014	28/06/2016	15	0.7%	19	1	19	1
Nevill Hall Hospital	V Lefemine*<br>F Soliman*	21/08/2014	31/03/2016	15	0.7%	19	1	22	1
York NHS Foundation Trust	J Piper*<br>E Baker<br>R Nasr*	21/11/2014	20/11/2015	15	0.7%	11	1	19	1

Hospital name	Local Collaborators	Date participant recruited		Total number recruited		Active recruitment months[1]		All recruitment months[2]	
		First	Last			Months actively recruiting	Monthly recruitment rate	Months actively recruiting	Monthly recruitment rate
**Overall**		**04/02/2014**	**30/06/2016**	**2108**		**28**	**75**	**28**	**75**
Kettering General Hospital	A Knight*<br>M Wright*<br>G Wilson*<br>S Musa*	24/09/2015	12/04/2016	14	0.7%	6	2	9	2
Peterborough City Hospital	E Popa*<br>S Goh*<br>G Shetty*	25/09/2014	12/03/2015	14	0.7%	5	3	21	1
Lancashire Teaching Hospitals Chorley	A Topps<br>N Bishop<br>G Boland*<br>Z Saidan*<br>B Murthy*	17/06/2014	21/10/2014	13	0.6%	4	3	24	1
St Helens and Knowsley NHS Trust	N Barnes<br>T Kiernan*<br>R Clifford<br>M Pennick<br>L Chagla*<br>A Iqbal*	29/05/2014	12/04/2016	13	0.6%	22	1	25	1
Countess of Chester Hospital	W Hamilton-Burke<br>H Lennon<br>C Harding-Mackean*	17/02/2015	24/11/2015	12	0.6%	9	1	16	1
County Durham and Darlington NHS Trust	T Collin*<br>J Henton	03/11/2014	23/05/2016	12	0.6%	18	1	19	1
Barnsley Hospital NHS Foundation Trust:	OS Olayinka<br>JR Dicks*	08/07/2015	15/06/2016	10	0.5%	11	1	11	1
Lincoln County Hospital	A Sami*<br>A Giaramadze*	19/01/2016	22/06/2016	10	0.5%	5	2	5	2
QE2 Welwyn Garden City	U Walsh<br>H Deol*	19/10/2015	08/02/2016	10	0.5%	3	3	8	1
Royal Glamorgan Hospital	S Datta<br>G Patel<br>C Steventon<br>R Foulkes*<br>E Vaughan Williams*<br>G Osborn*<br>C Gateley*	14/01/2016	11/04/2016	10	0.5%	2	5	5	2
Great Western Hospital, Swindon	I Dash<br>A Chaudry*<br>M Galea*<br>N Coombs*	09/06/2014	29/09/2014	10	0.5%	3	3	24	0
University Hospitals North Stafford	S Narayanan*<br>S Soumian*<br>V Voynov;	10/06/2014	02/09/2014	10	0.5%	2	5	24	0
Royal Bournemouth Hospital	E Skene*<br>C Laban*<br>A Iskender*<br>D Perry*	31/12/2015	16/06/2016	9	0.4%	5	2	5	2
Poole General Hospital	S Clark*<br>A Evans*	09/04/2015	24/06/2016	9	0.4%	14	1	14	1
Royal Surrey County Hospital	E Clayton<br>A Conway<br>T Irvine*<br>P Partlett*<br>F Pakzad	11/09/2014	17/03/2015	9	0.4%	6	2	21	0
Ashford and St Peters NHS Trust	R Newton<br>A Luangsomboon<br>B Swiech<br>A Robinson<br>M Runkel	30/06/2015	26/02/2016	8	0.4%	7	1	12	1
Burnley Hospital	A.Topps<br>M Amanita<br>S Gawne*<br>J McNicholas*<br>C Thomas<br>M Khanbhai<br>R Brindle<br>N Taheri<br>R Kuruvilla<br>M Saleh<br>F Bux<br>P Pugh<br>J Iddon	13/11/2014	22/02/2016	8	0.4%	15	1	19	0
Warrington and Halton Hospitals	N Sarfraz*<br>P Thawdar	11/06/2015	09/06/2016	8	0.4%	11	1	12	1
Chesterfield Royal Hospital	F Mazari<br>J Massey*<br>I Azmy*<br>C Hollywood*	16/02/2015	27/07/2015	6	0.3%	5	1	16	0
Prince Phillip Hospital	S Udayasankar<br>S Khawaja<br>Y Sharaiha*	19/01/2016	21/06/2016	6	0.3%	5	1	5	1
Yeovil District Hospital	N Dobner<br>C Osborne;	29/09/2014	18/04/2016	6	0.3%	18	0	21	0
Bedford Hospitals NHS Trust	N Manoloudakis<br>H Charfare<br>F Conroy*	19/11/2015	08/03/2016	5	0.2%	3	2	7	1
Craigavon Area Hospital	G Irwin<br>P Mallon*	06/10/2014	26/06/2015	5	0.2%	8	1	20	0
Altnagelvin Area Hospital	R Johnston*	09/12/2014	16/02/2015	4	0.2%	2	2	18	0
Dunedin Hospital	B Smith*	25/06/2015	17/09/2015	4	0.2%	2	2	12	0
East Surrey Hospital	E J Turner<br>S Waheed*	07/07/2015	08/09/2015	4	0.2%	2	2	11	0
University College Hospital, London	L Johnson<br>R Carpenter*<br>J Gattuso*<br>J Franks*	13/10/2014	29/01/2015	4	0.2%	3	1	20	0
Warwick Hospital	M Dakka<br>H Tafazal<br>L Jones*<br>D Enver*<br>D Clarke*<br>S Harries*	06/05/2015	09/06/2015	4	0.2%	1	4	13	0
Leighton Hospital	S Hignett*<br>V Pope	20/04/2016	28/06/2016	3	0.1%	2	2	2	2

Hospital name	Local Collaborators	Date participant recruited		Total number recruited		Active recruitment months[1]		All recruitment months[2]	
		First	Last			Months actively recruiting	Monthly recruitment rate	Months actively recruiting	Monthly recruitment rate
**Overall**		**04/02/2014**	**30/06/2016**	**2108**		**28**	**75**	**28**	**75**
Oxford University Hospitals NHS Foundation Trust	D Remoundos*<br>PJ Roy*	09/02/2016	09/05/2016	3	0.1%	3	1	4	1
University Hospital Southampton NHS Foundation Trust	S Robertson<br>R Cutress*	20/04/2015	13/07/2015	3	0.1%	2	2	14	0
Torbay and South Devon NHS Foundation Trust	C Ives<br>M Green*	12/10/2015	29/02/2016	3	0.1%	4	1	8	0
North Middlesex University Hospital	T Gandamihardja*	12/10/2015	03/12/2015	2	0.1%	1	2	8	0
Basingstoke and North Hampshire Hospital	V Kalles<br>K Harris*	20/06/2016	20/06/2016	1	0.1%	0	NA	0	NA
King George Hospital, Ilford	S Saha*<br>S Samlalsingh*<br>A Ogedegbe*	17/04/2015	17/04/2015	1	0.1%	0	NA	14	0
St Marys Hospital Isle of Wright	M Jobson*	07/10/2014	07/10/2014	1	0.1%	0	NA	20	0
L Barr (private)	L Barr*	26/11/2014	26/11/2014	1	0.1%	0	NA	19	0
Salisbury District Hospital	S Masood<br>V Brown*<br>G Murphy	14/04/2015	14/04/2015	1	0.1%	0	NA	14	0
St Georges Hospital	S Tang*<br>D Banerjee*	10/04/2015	10/04/2015	1	0.1%	0	NA	14	0

*Denotes consultant surgeon collaborator; A total 2108 eligible patients from 81 sites were recruited. Table sorted by total number recruited. [1]Calculated using the date of first recruited patient at site (or overall for overall row) and date of last recruited patient at site (or overall for overall row). [2]Calculated between the date of first recruited patient (or overall for overall row) and date last recruited patient in study.

Supplementary table 3. Detailed breakdown of key outcomes at 3 months by risk-factor

		Implant loss		Infection		Readmission		Reoperation	
	**Factor**	**Patients experiencing implant loss**	**Patients not experiencing implant loss**	**Patients treated for infection**	**Patients with no infection**	**Patients readmitted for complications**	**Patients not readmitted**	**Patients requiring reoperation for complications**	**Patients who did not require reoperation**
	N	182	1899	522	1559	372	1709	370	1711
**Patient risk factor**	**Age (years) (N)**	181	1890	518	1553	369	1702	367	1704
	MED (LQ - UQ)	51.0 (44.0-58.0)	49.0 (43.0-57.0)	50.0 (44.0-59.0)	49.0 (43.0-56.0)	49.0 (43.0-57.0)	50.0 (43.0-57.0)	49.0 (43.0-57.0)	50.0 (43.0-57.0)
	(MIN, MAX)	(16.0, 80.0)	(19.0, 83.0)	(19.0, 80.0)	(16.0, 83.0)	(19.0, 83.0)	(16.0, 81.0)	(19.0, 83.0)	(16.0, 81.0)
	Not known	1	9	4	6	3	7	3	7
	**Smoking**								
	Non/ex smoker	150 (84%)	1687 (90%)	449 (88%)	1388 (90%)	315 (86%)	1522 (90%)	312 (86%)	1525 (90%)
	Current smoker/nicotine replacement	29 (16%)	185 (10%)	64 (12%)	150 (10%)	52 (14%)	162 (10%)	51 (14%)	163 (10%)
	Not known	3	27	9	21	5	25	7	23
	**BMI (kg/m^2) (N)**	171	1792	500	1463	345	1618	344	1619
	MED (LQ - UQ)	26.1 (23.8-30.9)	24.7 (22.2-28.0)	26.4 (23.6-30.5)	24.3 (22.0-27.6)	26.0 (23.5-29.9)	24.6 (22.0-28.0)	25.4 (23.0-29.1)	24.6 (22.1-28.0)
	(MIN, MAX)	(15.0, 44.5)	(14.4, 54.0)	(15.0, 44.5)	(14.4, 54.0)	(15.0,44.4)	(14.4, 54.0)	(15.0,42.6)	(14.4, 54.0)
	Not known	11	107	22	96	27	91	26	92
	**Previous radiotherapy**								
	Yes	21 (12%)	115 (6%)	52 (10%)	84 (5%)	33 (9%)	103 (6%)	33 (9%)	103 (6%)
	No	161 (88%)	1771 (94%)	467 (90%)	1465 (95%)	336 (91%)	1596 (94%)	334 (91%)	1598 (94%)
	Not known	0	13	3	10	3	10	3	10
	**Neoadjuvant chemo**	13 (7%)	210 (11%)	44 (9%)	179 (12%)	36 (10%)	187 (11%)	32 (9%)	191 (11%)
	Yes	165 (93%)	1667 (89%)	470 (91%)	1362 (88%)	329 (90%)	1503 (89%)	330 (91%)	1502 (89%)
	No	4	22	8	18	7	19	8	18
	Not known								
**Operative risk factor**	**Length of operation (minutes) (N)**	165	1727	473	1419	338	1554	342	1550
	MED (LQ - UQ)	180.0 (150.0-227.0)	180.0 (147.0-210.0)	180 (150.0-220.0)	180.0 (146.0-210.0)	180.0 (150.0-223.0)	180.0 (146.0-210.0)	180.0 (150-225.0)	180.0 (146.0-210.0)
	(MIN, MAX)	(85.0, 570.0)	(60.0, 480.0)	(75.0, 570.0)	(60.0, 480.0)	(75.0, 570.0)	(60.0, 480.0)	(70.0, 570.0)	(60.0, 480.0)
	Not known	17	172	49	140	34	155	28	161
	**Bilateral surgery**								
	Yes	59 (32%)	482 (25%)	158 (30%)	383 (25%)	118 (32%)	423 (25%)	121 (33%)	420 (25%)
	No	123 (68%)	1417 (75%)	364 (70%)	1176 (75%)	254 (68%)	1286 (75%)	249 (67%)	1291 (75%)
	Not known	0	0	0	0	0	0	0	0
	**Nipple sparing mastectomy**								
	Yes	47 (26%)	433 (23%)	115 (22%)	365 (24%)	93 (25%)	387 (23%)	100 (27%)	380 (22%)
	No	131 (72%)	1439 (76%)	398 (77%)	1172 (76%)	272 (74%)	1298 (76%)	263 (71%)	1307 (77%)
	Different approach per breast	3 (2%)	18 (1%)	6 (1%)	15 (1%)	5 (1%)	16 (1%)	5 (1%)	16 (1%)
	Not known	2	8	2	8	2	8	2	8
	**Risk reducing vs. therapeutic mastectomy**								
	Risk reducing only	33 (18%)	374 (20%)	112 (21%)	295 (19%)	81 (22%)	326 (19%)	82 (22%)	325 (19%)
	Therapeutic only	126 (69%)	1357 (72%)	360 (69%)	1123 (72%)	249 (67%)	1234 (72%)	243 (66%)	1240 (73%)
	Different approach per breast	23 (13%)	164 (9%)	50 (10%)	137 (9%)	42 (11%)	145 (9%)	45 (12%)	142 (8%)
	Not known	0	4	0	4	0	4	0	4
	**Fixed vs. adjustable implants**								
	Fixed	102 (56%)	1118 (59%)	287 (55%)	933 (60%)	209 (57%)	1011 (60%)	211 (58%)	1009 (59%)
	Adjustable	79 (44%)	761 (40%)	230 (44%)	610 (39%)	159 (43%)	681 (40%)	155 (42%)	685 (40%)
	Different approach per breast	0 (0%)	4 (0%)	1 (0%)	3 (0%)	0 (0%)	4 (0%)	0 (0%)	4 (0%)
	Not known	4	13	4	13	4	13	4	13

Percentages are calculated using the total number of patients with data available (*N)* for that column; LQ – lower quartile; Med – median, Min – minimum, Max – maximum; UQ – upper quartile.
